# Kaposi’s sarcoma-associated herpesvirus (KSHV) gB dictates a low-pH endocytotic entry pathway as revealed by a dual-fluorescent virus system and a rhesus monkey rhadinovirus expressing KSHV gB

**DOI:** 10.1371/journal.ppat.1012846

**Published:** 2025-01-16

**Authors:** Shanchuan Liu, Sarah Schlagowski, Anna K. Großkopf, Natalia Khizanishvili, Xiaoliang Yang, Scott W. Wong, Elina M. Guzmán, Marija Backovic, Stefano Scribano, Arne Cordsmeier, Armin Ensser, Alexander S. Hahn

**Affiliations:** 1 Junior Research Group Herpesviruses, Infection Biology Unit, German Primate Center–Leibniz Institute for Primate Research, Göttingen, Germany; 2 Vaccine and Gene Therapy Institute, Oregon Health & Science University, Beaverton, OR, United States of America; 3 Institut Pasteur, Université Paris Cité, CNRS UMR3569, Unité de Virologie Structurale, Paris, France; 4 Friedrich-Alexander Universität Erlangen-Nürnberg, Erlangen, Germany; Harvard University, UNITED STATES OF AMERICA

## Abstract

Interaction with host cell receptors initiates internalization of Kaposi’s sarcoma-associated herpesvirus (KSHV) particles. Fusion of viral and host cell membranes, which is followed by release of the viral capsid into the cytoplasm, is executed by the core fusion machinery composed of glycoproteins H (gH), L (gL), and B (gB), that is common to all herpesviruses. KSHV infection has been shown to be sensitive to inhibitors of vacuolar acidification, suggestive of low pH as a fusion trigger. To analyze KSHV entry at the single particle level we developed dual-fluorescent recombinant KSHV strains that incorporate fluorescent protein-tagged glycoproteins and capsid proteins. In addition, we generated a hybrid rhesus monkey rhadinovirus (RRV) that expresses KSHV gB in place of RRV gB to analyze gB-dependent differences in infection pathways. We demonstrated lytic reactivation and infectivity of dual-fluorescent KSHV. Confocal microscopy was used to quantify co-localization of fluorescently-tagged glycoproteins and capsid proteins. Using the ratio of dual-positive KSHV particles to single-positive capsids as an indicator of fusion events we established KSHV fusion kinetics upon infection of different target cells with marked differences in the “time-to-fusion” between cell types. Inhibition of vesicle acidification prevented KSHV particle-cell fusion, implicating low vesicle pH as a requirement. These findings were corroborated by comparison of RRV-YFP wildtype reporter virus and RRV-YFP encoding KSHV gB in place of RRV gB. While RRV wt infection of receptor-overexpressing cells was unaffected by inhibition of vesicle acidification, RRV-YFP expressing KSHV gB was sensitive to Bafilomycin A1, an inhibitor of vacuolar acidification. Single- and dual-fluorescent KSHV strains eliminate the need for virus-specific antibodies and enable the tracking of single viral particles during entry and fusion. Together with a hybrid RRV expressing KSHV gB and classical fusion assays, these novel tools identify low vesicle pH as an endocytotic trigger for KSHV membrane fusion.

## Author summary

All herpesviruses encode a fusion protein, gB, which fuses the virus membrane with host membranes. Membrane fusion is essential for infection by enveloped viruses. Under conditions where gB proteins from other herpesviruses fuse cells into syncytia, KSHV gB does not readily fuse cells, and KSHV infection was previously shown to be inhibited by substances that raise the pH of endocytotic vesicles. We therefore sought to test whether fusion of KSHV viral particles with cellular membranes is a pH-dependent step. We developed two tools to test this hypothesis. The first tool is a dual-fluorescent KSHV with differently colored protein tags at the viral envelope and the capsid, which lose colocalization upon fusion and release of the capsid from the membrane, but not in the presence of an inhibitor of vesicle acidification. The second tool is a hybrid RRV strain that expresses KSHV gB instead of RRV gB. RRV infected receptor-overexpressing cells in a manner that was not inhibited by an inhibitor of vesicle acidification. The hybrid RRV expressing KSHV gB on the other hand was sensitive to inhibition of vesicle acidification. Together, these findings show that KSHV gB dictates a low-pH, endocytotic route of infection.

## Introduction

Kaposi’s sarcoma-associated herpesvirus (KSHV) is a human oncogenic herpesvirus. It is associated with several malignancies: Kaposi’s sarcoma, multicentric Castleman’s disease, primary effusion lymphoma, and potentially osteosarcoma [1] (reviewed in [2]). It is further associated with Kaposi Sarcoma inflammatory cytokine syndrome (KICS) [3,4]. KSHV is associated with a substantial burden of disease in Sub-Saharan Africa and in at-risk populations, such as men who have sex with men or immunocompromised individuals worldwide (reviewed in [2]). Its primary route of transmission is believed to be saliva exchange, including mother-to-child transmission in Sub-Sahara Africa [5].

**Fig 1:**
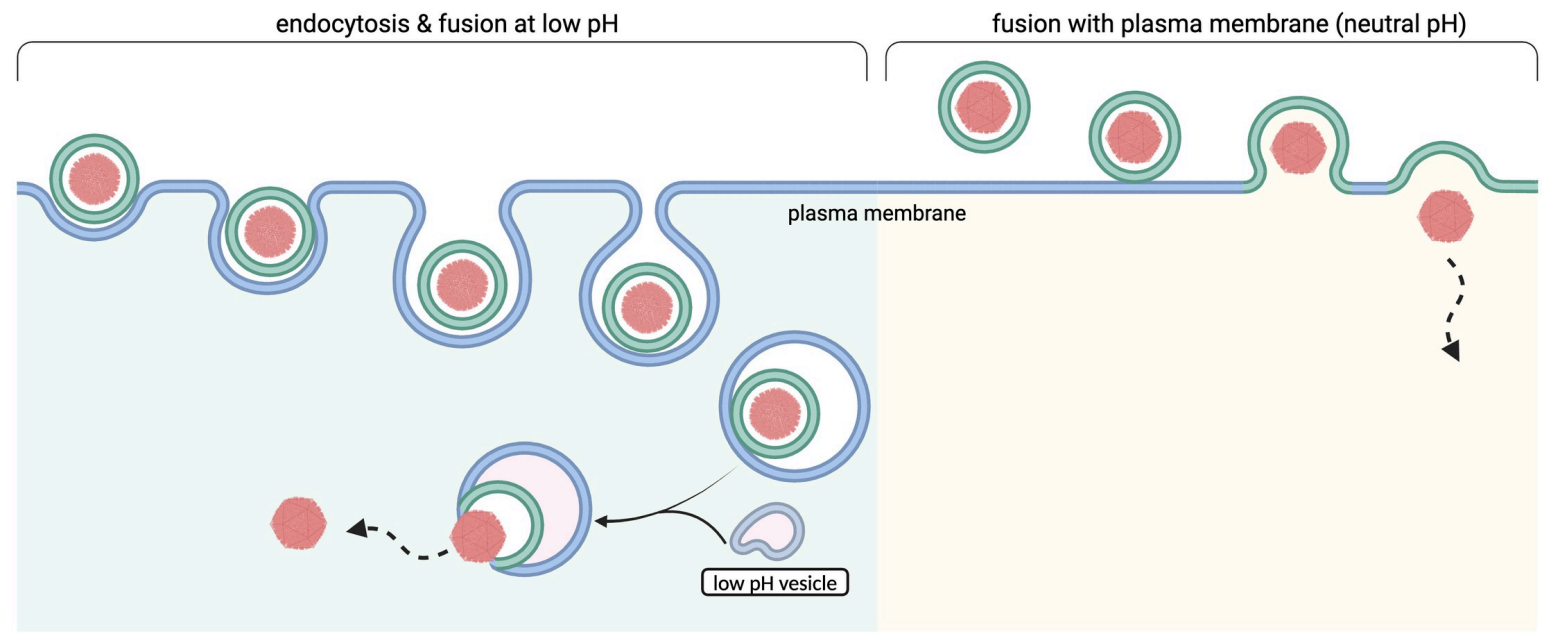
Schematic illustration of endocytotic infection by enveloped viruses involving low pH versus direct fusion at the plasma membrane (created using biorender.com).

Enveloped viruses including the herpesviruses employ two different strategies regarding the timing of membrane fusion: they either fuse directly with the plasma membrane at the cell surface or they fuse from the interior of endocytotic vesicles with the membranes of these vesicles (Fig 1). For the first strategy, endocytosis is not necessary and potentially detrimental to infection if the environment in endocytotic compartments is not conducive for fusion. For the second strategy, endocytosis is a prerequisite. As in most biological systems the distinction is usually not absolute and many viruses are capable of utilizing both strategies, but they usually fall predominantly into one or the other category. Often, the ability of viral glycoproteins to efficiently cause cell-cell fusion upon expression correlates well with the ability of the respective viruses to enter through fusion at the plasma membrane. An instructive example is SARS-CoV-2, which even oscillates depending on the variant and its Spike protein between the two extremes. While the Delta variant Spike induces syncytia and mediates infection primarily through direct fusion, the BA.1 variant Spike does not induce syncytia and mediates entry primarily through endocytosis [6], at least in experimental systems.

Among the herpesviruses, herpes simplex virus is a well-studied example of a virus with highly fusogenic glycoproteins that predominantly employs the direct fusion strategy but can also use the endocytotic route in some circumstances, potentially through pH-induced conformational changes in gB [7–10]. The human cytomegalovirus is also capable of using both strategies, depending on the target cell [11–13]. KSHV, on the other hand, seems to fall into the second category, with many reports describing the importance of endocytotic pathways for infection [14–20]. Similar data exist for the related murid gammaherpesvirus 4 strain, murine gammaherpesvirus 68 (MHV-68) [21], whereas the Epstein-Barr virus (EBV) infects at least epithelial cells and Raji lymphoblastoid cells via direct membrane fusion, and B cells through an endocytotic pathway, both routes likely pH-independent [22].

KSHV uses a number of receptors for attachment and entry. It binds to cells through heparan sulfate proteoglycans, such as syndecans, which are engaged by glycoproteins K8.1, gB, KCP and gH [23–27]. gB additionally interacts with integrins [28,29], neuropilin-1 [15], and DC-SIGN [30,31]. Integrins, neuropilin-1, and potentially DC-SIGN further promote endocytosis and/or macropinocytosis [15,32]. KSHV also interacts with members of the Eph family of receptor tyrosine kinases, primarily with EphA2, through the gH/gL glycoprotein complex at a post-attachment step, and this interaction promotes endocytosis and/or macropinocytosis and fusion [14,16,19,33–39]. It has been observed that KSHV does not readily induce cell-cell fusion. While some groups managed to obtain a weak signal in cell-cell fusion assays [15,40,41], others did not [35,42]. Generally, the cell-cell fusion activity of KSHV glycoproteins is far below that of other viral glycoproteins, even from closely related viruses like the Epstein-Barr virus (EBV) [35] or the rhesus monkey rhadinovirus (RRV) [42,43]. These observations align well with several publications that describe endo- or macropinocytotic entry of KSHV into target cells [14–20], and with reports describing inhibition of infection by substances that inhibit vesicle acidification [20,33,42,44]. An open question remains whether the inhibition of vesicle acidification has a direct effect on fusion of the viral and host cell membranes or whether it disrupts other processes like particle trafficking or post-entry signaling. While Gillet *et al*. described a change in antigenicity of MHV-68 gB that was dependent on vesicle acidification [21], it remains unclear whether such requirements for low vesicle pH directly reflected pH-sensitivity of gB itself or of any of the other factors that govern gB’s activation, and whether such findings translate to KSHV gB-mediated virus-cell fusion.

We hypothesized that KSHV gB exhibits low fusogenicity at neutral pH and needs acidification of endocytotic vesicles for initiation of the fusion process. To test this hypothesis, we applied two approaches: i) we generated a dual-fluorescent KSHV that allows monitoring the separation of viral envelope proteins and capsid as a surrogate marker for fusion, and ii) we generated a hybrid RRV carrying the gB of KSHV instead of its own.

## Results

### KSHV gB exhibits low fusogenicity

**Fig 2:**
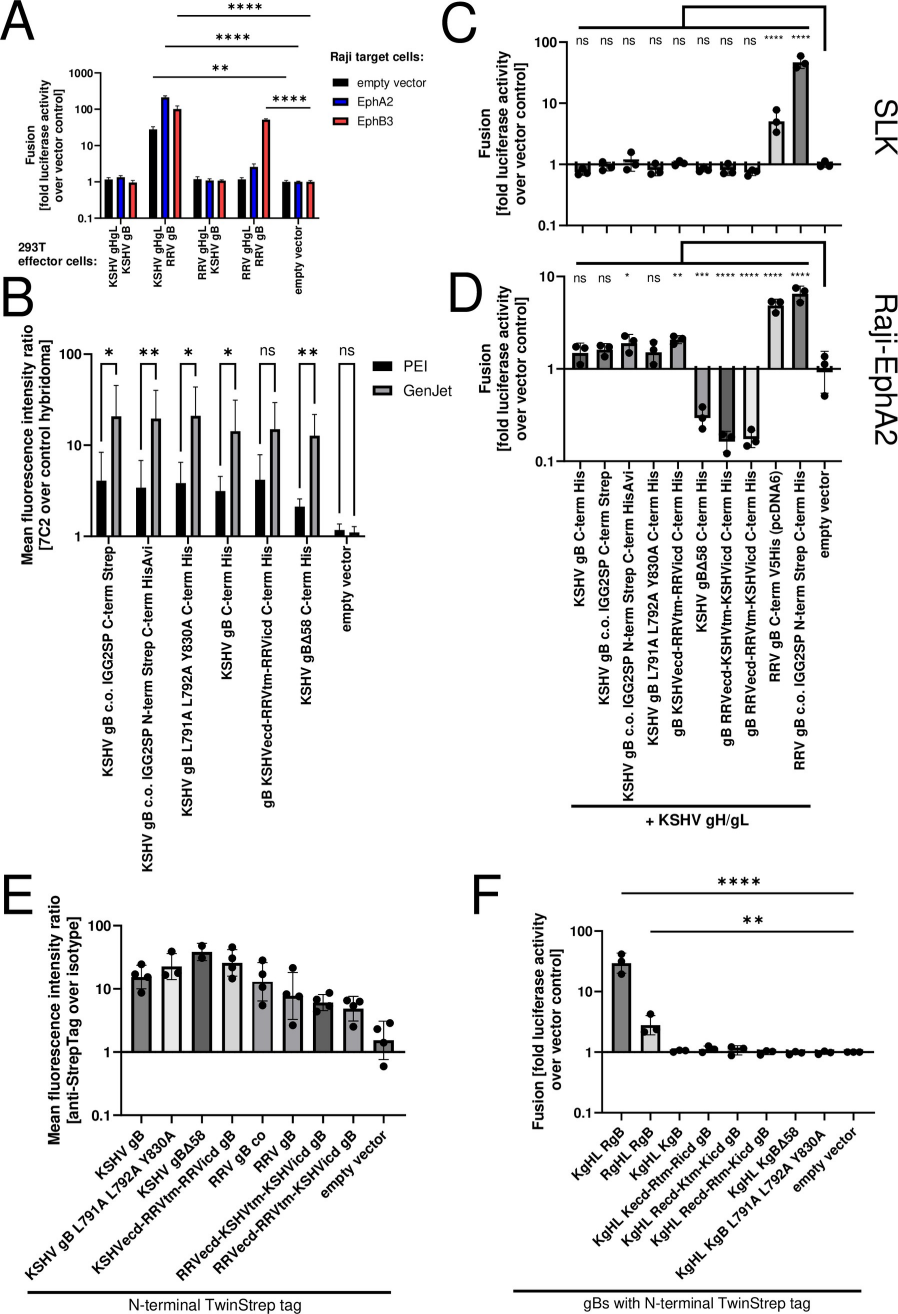
Low fusion activity of KSHV gB in cell-cell fusion assays despite cell surface expression. A) Raji target cells were transduced with expression constructs for the indicated receptor proteins and were co-cultured with HEK293T effector cells transfected with expression constructs for the indicated viral glycoproteins or an empty vector control. Fusion was quantified by luciferase readout and normalized using the empty vector control (no viral glycoproteins) for each target cell population. Error bars represent the standard deviation, the experiment was performed in triplicates. Only significant differences from the empty vector (no viral glycoproteins) effector cell control are shown. * p<0.05; ** p<0.01; **** p<0.0001; two-way ANOVA, Dunnett test with family-wise correction for multiple comparison. All values were log-transformed prior to analysis. B) Cell-surface expression of KSHV gB driven by different expression constructs and two different transfection protocols. HEK293T cells were transfected with indicated pCAGGS-based gB constructs using either PEI or GenJet as transfection agent. ns non significant; * p<0.05; ** p<0.01; two-way ANOVA, Sidak test for multiple comparison. All values were log-transformed prior to analysis. ecd: extracellular domain; tm: transmembrane domain; icd: intracellular domain; N-term: N-terminal; C-term: C-terminal. C) SLK target cells or D) Raji-EphA2 target cells as in (A) were co-cultured with HEK293T effector cells transfected with expression constructs for the indicated viral glycoproteins or an empty vector control. Fusion was quantified by luciferase readout and normalized using the empty vector control (no viral glycoproteins) for each target cell population. Error bars represent the standard deviation, the experiment was performed in triplicates. ns non significant; * p<0.05; ** p<0.01; *** p<0.001, **** p<0.0001; ordinary one-way ANOVA, Dunnett test with family-wise correction for multiple comparison. All values were log-transformed prior to analysis. Strep: TwinStrep tag; His: Polyhistidine tag; V5: V5 epitope tag; IGG2SP: IgG2 signal peptide; Avi: Avi tag. E) HEK293T cells were transfected with the indicated constructs bearing an N-terminal TwinStrep tag and cell surface expression was quantified by flow cytometry. All values were log-transformed prior to analysis. F) Fusion assay with HEK293T effector cells transfected to express the indicated constructs and SLK target cells was performed as in (C). Error bars represent the standard deviation, the experiment was performed three times in triplicates. ns non significant; ** p<0.01;**** p<0.0001; ordinary one-way ANOVA, Dunnett test for multiple comparison. All values were log-transformed prior to analysis. K KSHV; R RRV.

Cell-cell fusion assays confirmed the extremely low intrinsic fusogenicity of KSHV gB which is either non-existent at neutral pH or at least orders of magnitude below that of RRV gB (Fig 2A). As in previous studies [35,42,45], KSHV gH/gL activated the heterologous RRV gB potently, but not KSHV gB. As low fusion activity can be the result of low surface expression, we generated and tested a number of different KSHV gB constructs, including a construct with mutated endocytosis motifs (L791A L792A, a dileucine motif, and Y830A, a YXXphi motif) [46]. In addition, we used a different vector backbone. These constructs were expressed but did not exhibit fusion activity in our assay (S1A and S1B Fig). We also generated a construct deleted in the 58 amino acids from the C-terminus of gB, which according to two studies allows for cell-cell fusion activity [40,47]. This deletion removes 62% of the intracellular domain and the resulting protein might not be representative of the original protein anymore. All these KSHV gB constructs were cloned in the pCAGGS vector [48] and exhibited considerably more robust cell surface expression compared to pcDNA-based constructs as judged by cell-surface staining with a newly developed KSHV gB monoclonal antibody (S1C Fig). Interestingly, apart from the choice of the vector backbone none of the different modifications, namely mutation of endocytosis motifs, partial deletion of the intracellular domain, swapping the intracellular domains with those of RRV gB, replacing the signal peptide with an IgG2 signal peptide or optimizing the codon usage influenced cell-surface expression levels of KSHV gB appreciably (S1C Fig).

To further optimize the sensitivity of our fusion assay, after having optimized the choice of the vector backbone to boost expression of KSHV gB, we compared two transfection methods and found the GenJet reagent to result in higher expression than polyethylenimine (PEI) transfection for all tested constructs, again without revealing any obvious differences in surface expression between different KSHV gB constructs (Fig 2B). We then switched to using GenJet transfection agent for fusion assays. Under these optimized conditions, none of the KSHV gB constructs coexpressed with KSHV gH and gL exhibited cell-cell fusion activity at a level significantly different from background with SLK target cells, which are highly susceptible to KSHV infection and express the gH/gL receptor EphA2 [34], while RRV gB, as expected, readily mediated cell-cell fusion (Fig 2C). The same set of gB constructs were also tested together with KSHV gH/gL using EphA2-overexpressing Raji cells as targets and using the GenJet transfection method (Fig 2D). Here, we found that two out of six KSHV constructs (those containing the KSHV ectodomain) exhibited significant but low fusion activity, only approximately twofold over background and with fluctuation between constructs, overall not supporting robust fusion activity. The strong overexpression may have resulted in toxicity for some of proteins, as some constructs exhibited reporter activity below background levels and observed activity for RRV gB was numerically lower than in our previous assays with slightly less potent overexpression.

Next, we generated a number of expression constructs encoding gBs that are chimeric between RRV and KSHV. Replacing the RRV gB intracellular domain (ICD) with that of KSHV led to a total loss of fusion activity, implicating the intracellular domain as a regulator of fusion (Figs 2C, 2D and S2A). To our surprise we found that swapping the domains in either direction resulted in a total (SLK target cells Fig 2C) or near total (Raji-EphA2 target cells, Figs 2D and S2A) loss of fusion activity in our assays. We therefore concluded that the mechanisms controlling the activity of these proteins are not dictated by the intracellular domain alone, are likely not encoded in a single domain, and require a more detailed, ideally structure-guided analysis.

The possibility remained that the observed low fusogenicity of KSHV gB, even when expressed on the cell surface, was due to relatively lower cell surface expression compared to RRV gB. To directly compare cell surface expression between the two different gBs, we added an N-terminal TwinStrep-tag (abbreviated as Strep for brevity in the legend) and heterologous signal peptide, which does not appreciably alter surface expression of KSHV gB (Figs S1C and 2B), to the gB constructs and measured cell surface expression (Fig 2E) and cell-cell fusion with SLK target cells (Fig 2F). Clearly, the KSHV gB constructs were expressed at high levels on transfected 293T cells. Expression levels were comparable to if not numerically higher than the expression levels achieved with the different RRV gB constructs, but the KSHV gB constructs still did not show appreciable fusion activity with SLK target cells whereas RRV gB did when coexpressed with KSHV gH/gL (Fig 2F).

These results support the notion that virus-cell fusion of KSHV is best studied using a viral system, such as dual-fluorescent KSHV or RRV with KSHV hybrid glycoproteins, rather than cell-cell fusion assays which for KSHV only allow for low sensitivity and small effect sizes in comparison to other viral fusion proteins.

### Dual-fluorescent KSHV is replication-competent

**Fig 3:**
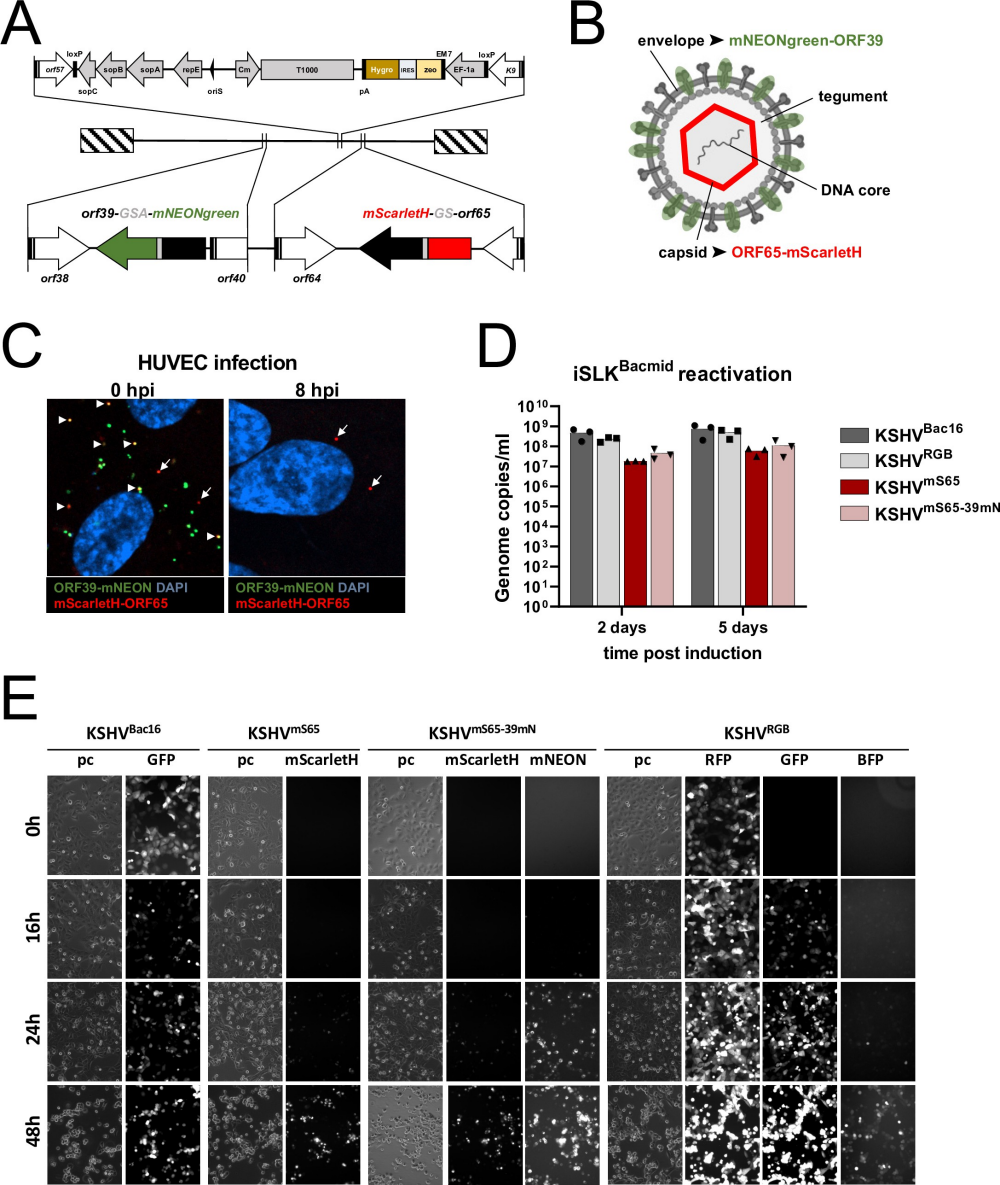
Construction of dual-fluorescent KSHV. A) Map of BAC 16 noGFP_mScarletH-ORF65_ORF39-mNeonGreen encoding KSHV^mS65-39mN^ (not exactly to scale). B) Schematic drawing to illustrate the composition of dual-labeled KSHV particles (not to scale). C) HUVEC cells imaged after virus attachment in the cold 0 hours post infection (hpi) or after incubation at 37°C at 8 hpi. Arrow heads indicate colocalization of mScarletH-ORF65 and ORF39-mNeonGreen, arrows indicate single positive capsids. hpi: hours post infection. D) Release of encapsidated genomes after chemical induction of the lytic cycle in iSLK producer cells harboring the indicated viruses. E) Fluorescence microscopy of iSLK cells that were infected with the first passage of the indicated viruses, selected and subjected to chemical induction of the lytic cycle. The cells were imaged in culture without fixation at the indicated timepoints after induction of the lytic cycle.

We previously reported a KSHV reporter virus with an N-terminal mNeonGreen added to the capsid protein ORF65, which had resulted in replication-competent virus [42], that can be tracked on a single particle level without the need for virus-specific antibodies. Based on this approach we constructed virus mutants harboring either an N-terminal mScarletH-tagged ORF65 alone or in combination with mNeonGreen at the C-terminus of ORF39, which encodes glycoprotein M (gM) (Fig 3A), similar to a system that had been developed for the human cytomegalovirus [49].

This resulted in dual-fluorescent viruses, labelled at the envelope through gM and at the capsid through ORF65 (Fig 3B). As expected, when imaged directly after addition to HUVEC cells, most red particles representing viral capsids were colocalized with green signal representing viral envelope. Of note, we detected a large number of single-mNeonGreen(gM)-positive spots without associated mScarletH signal (S3 Fig). These gM-positive spots, which may represent “empty” virions that do not include a capsid, membrane or exocytotic vesicles or simply cell debris, do not contribute to the calculation of gM-colocalized capsid. By 8h post infection mostly red capsids were visible, devoid of the green virus envelope. When we compared reactivation and release from producer cells of these tagged viruses to that of BAC16 or KSHV BAC16 RGB based viruses [50], virus yield was reduced on the order of one log10 by addition of mScarletH to ORF65. Introducing mNeonGreen fused to gM did not further impact virus yield (Fig 3D). The virus particles were infectious and fully replication competent as proven by another passage of the released virus and subsequent reactivation (Fig 3E). As the idea was to study fusion events and not replication over time, we decided to accept a reduction in virus yield, which probably is to be expected when adding several hundred copies, according to the literature between 800 and 960 copies [51], of a fluorescent protein to the virus capsid. We proceeded to use these viruses to study the fusion of KSHV with cellular membranes during entry into target cells.

### Fusion kinetics differ between different cells

**Fig 4:**
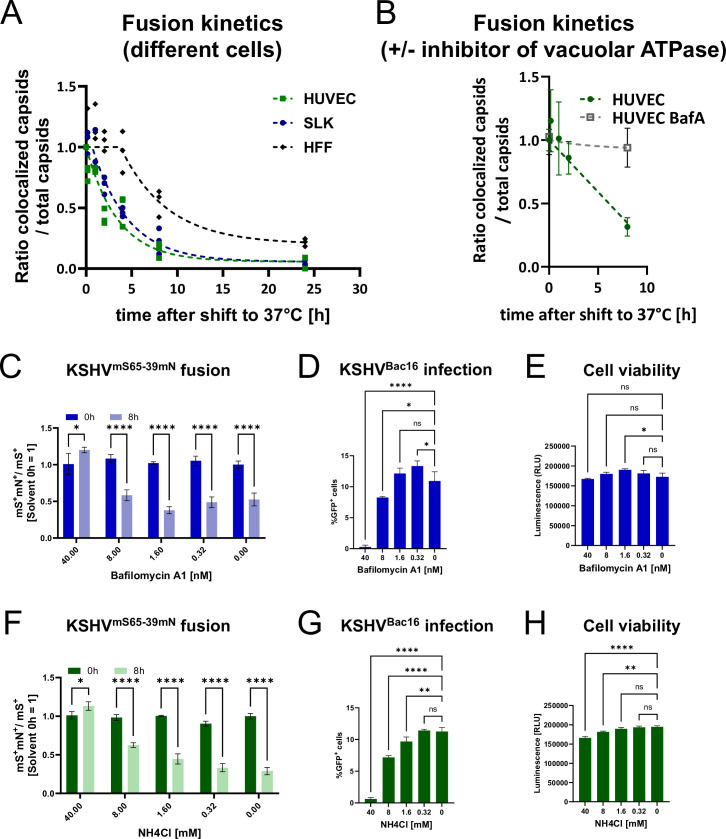
Cell-specific fusion kinetics and inhibition of fusion by inhibitors of vesicle acidification. A) Kinetics of virus-cell fusion with KSHV^mS65-39mN^ were plotted for HFF, HUVEC and SLK cells. The 0-hour-timepoint was set to 1 for all cells. The “Dissociation–One phase exponential decay” (HUVEC) or “Plateau followed by one phase decay” (SLK, HFF) functions of GraphPad PRISM were used for non-linear curve fitting, and the model with better fit was chosen (Y0 set to 1; R squared greater than 0.85 for all models). Data represent three independently produced virus stocks (represented by individual dots). B) Virus fusion with HUVEC was analyzed as in (A) but in the absence or presence of Bafilomycin A1 (BafA). Error bars represent the standard deviation from three different images. C) Virus-cell fusion with SLK was analyzed as in (A) at the indicated timepoints and in the absence or presence of the indicated concentrations of Bafilomycin A1. D) SLK cells were infected with KSHV BAC 16 in the presence of the indicated concentrations of Bafilomycin A1 and infection was quantified by flow cytometry measuring the expression of the GFP reporter gene. E) SLK cells were incubated with Bafilomycin A1 and viability was measured. F) Virus-cell fusion with SLK was analyzed as in (C) in the presence of the indicated concentrations of ammonium chloride (NH4Cl). G) SLK cells were infected as in (D) in the presence of the indicated concentrations of ammonium chloride (NH4Cl). H) SLK cell viability was measured as in (E). Error bars represent the standard deviation. ns non-significant; * p<0.05; ** p<0.01; **** p<0.0001; n = 3 for C-H; two-way ANOVA with Sidak’s correction for multiple comparison (C, F), ordinary one-way ANOVA with Dunnett test for multiple comparison (D, E, G, H).

We first analyzed the KSHV fusion kinetics in different types of cells. We performed time-lapse experiments to assess the time it would take for the envelope signal to dissociate from the capsid, a surrogate marker for virus-cell fusion. We chose SLK cells, an epithelial cell line, human umbilical vein endothelial cells (HUVEC), and human foreskin fibroblasts (HFF). We found that KSHV exhibited different fusion kinetics when infecting the different cells. Fusion occurred fastest in HUVEC and SLK cells, and considerably more slowly in HFF cells (Fig 4A).

### KSHV virus-cell fusion is sensitive to inhibitors of vesicle acidification

We next tested whether virus-cell fusion would be affected by inhibition of vesicle acidification. Our hypothesis was that the inhibitory activity of these substances against infection was based on their effect on virus-cell fusion. Indeed, we found that Bafilomycin A1, an inhibitor of vacuolar ATPase [52], inhibited separation of envelope and capsids in HUVEC and SLK cells (Fig 4B and 4C) and, as expected, also inhibited KSHV infection of SLK cells (Fig 4D) without toxicity (Fig 4E). Similar results were obtained with ammonium chloride (NH4Cl), a lysosomotropic agent that raises endosomal pH [53], which also prevented separation of capsid and envelope proteins (Fig 4F), inhibited infection (Fig 4G), but exhibited mild toxicity (Fig 4H) in SLK cells.

We also controlled for the possibility that one of the two fluorescent proteins loses fluorescence more quickly under conditions of low pH, which would then result in an apparent loss of colocalization in case gM-mNeonGreen would fade faster than mScarletH-ORF65 in the capsid. When switching the position of the two fluorescent proteins (labelling gM with mScarletH and ORF65 with mNeonGreen), we observed the same separation of envelope proteins and capsid over time, and this again was inhibited by the addition of Bafilomycin A1 (S4 Fig).

### RRV carrying KSHV gB loses the ability to bypass inhibition by Bafilomycin A1 and depends on vesicle acidification for infection

**Fig 5:**
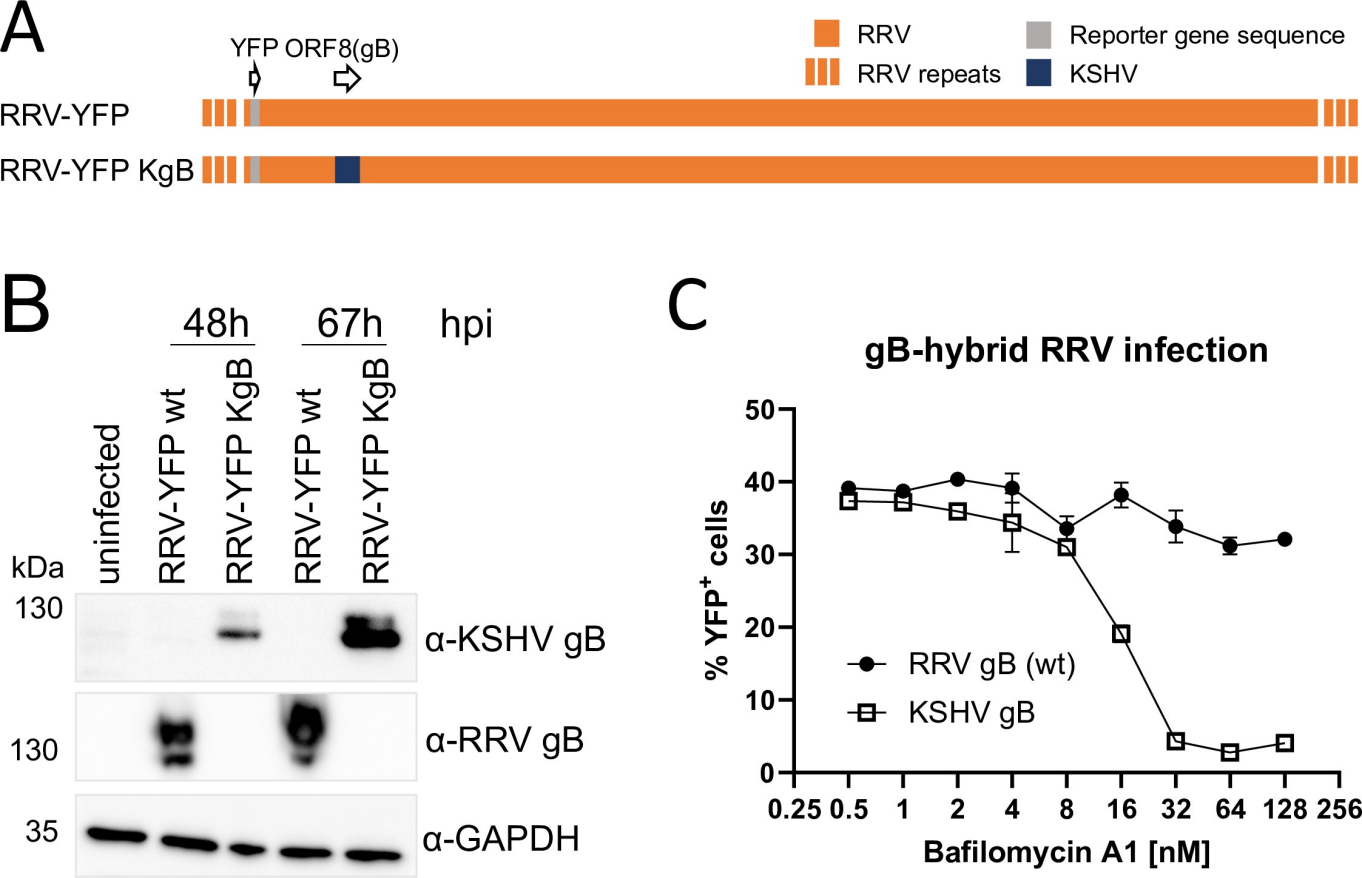
KSHV gB dictates sensitivity to inhibition of vesicle acidification. A) Schematic illustration of RRV-YFP KgB. B) Western blot analysis of lysates from rhesus monkey fibroblasts, which are permissive for lytic RRV replication and were infected with RRV-YPF or RRV-YFP KgB. The cells were harvested for analysis at the indicated timepoints (hpi: hours post infection). C) Raji cells that had been transduced to express the RRV gH/gL receptor EphB3 were infected with RRV-YFP with wt gB or with RRV-YFP KgB expressing KSHV gB instead of RRV gB. Infection was carried out in the presence of the indicated concentrations of Bafilomycin A1 and quantified as the percentage of YFP^+^ cells at 48 hpi. The experiment was performed in triplicate, error bars represent the standard deviation.

RRV infection is to a large extent sensitive to inhibitors of vesicle acidification [33], but RRV’s core fusion machinery is considerably more fusogenic in cell-cell fusion assays than that of KSHV (Fig 2). Therefore, we wanted to test whether RRV could bypass the requirement for vesicle acidification under conditions of high receptor expression, and whether this is dependent on gB. To answer this question, we constructed an RRV that expresses KSHV gB instead of its own gB, RRV-YFP KgB (Fig 5A and 5B). As the gH, gL, and gB glycoproteins of KSHV and RRV (Fig 2) or also EBV [35] function together in cell-cell fusion assays we expected the virus to be replication competent, which was the case. Earlier studies demonstrated that RRV infection of several different cell lines and primary cells occurs through endocytosis and is to a considerable degree sensitive to Bafilomycin A1 [33,54]. On the other hand, RRV gH, gL, and gB readily effect cell-cell fusion when expressed together [43]. We therefore speculated that recombinant overexpression of the EphB3 receptor by means of a lentiviral vector might suffice to enable RRV to infect independently of acidification through fusion at the plasma membrane. Indeed, when Raji cells were engineered to express high amounts of the fusion receptor EphB3 through lentiviral transduction (S5A Fig), RRV infection occurred independently of vesicle acidification as demonstrated by resistance to Bafilomycin A1 treatment (Fig 5C, filled circles). Interestingly, we also found RRV infection of non-transduced Raji cells to be largely insensitive to Bafilomycin A1 (S5B Fig). Therefore, Raji seem to be infected by RRV in a manner that is predominantly independent of vacuolar ATPase activity. Nevertheless, EphB3 overexpression confers high susceptibility as opposed to unmodified Raji cells, where no more than approx. 5% infected cells were achievable with our virus stocks (S5B Fig). When we tested RRV-YFP KgB on EphB3-overexpressing Raji cells, this KSHV gB chimeric virus had lost the ability to infect independently of vesicle acidification compared to RRV-YFP wt and was susceptible to Bafilomycin A1-mediated inhibition (Fig 5C, open boxes).

### KSHV infection in the presence of an inhibitor of vacuolar acidification can be rescued by acidification after virus binding

**Fig 6:**
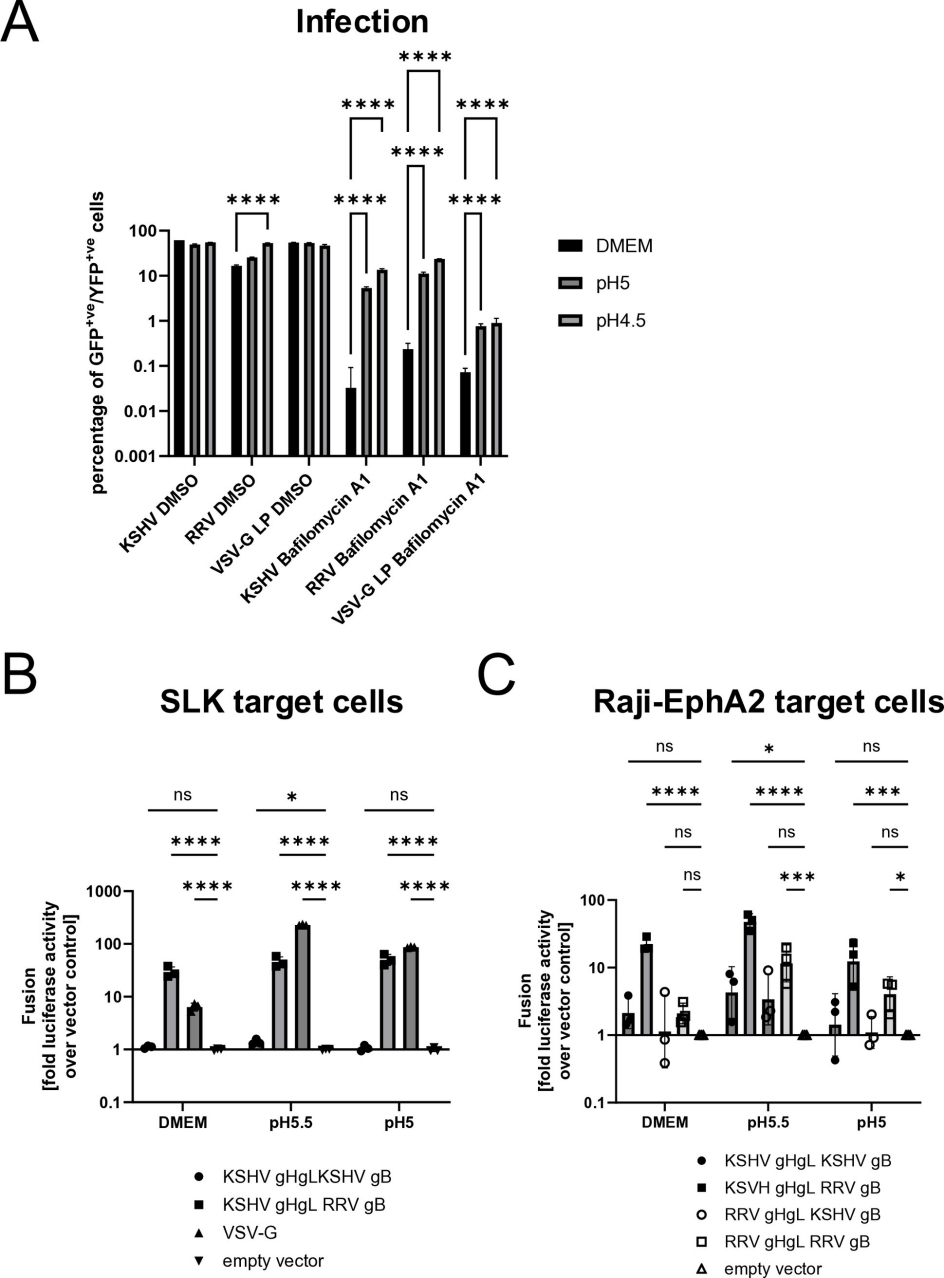
KSHV infection and fusion are driven by low pH. A) KSHV infection can proceed in the presence of an inhibitor of vacuolar acidification if pH is lowered exogenously. KSHV was allowed to adsorb to SLK cells, which were treated with Bafilomycin A1 or solvent control, in the cold. pH was then lowered by addition of citric acid-buffered medium and infection was allowed to proceed at 37°C for 30min, then pH was restored to normal. Error bars represent the standard deviation. ns non-significant; **** p<0.0001; the experiment was performed in triplicate and is representative for several similar experiments; two-way ANOVA with Dunnett’s correction for multiple comparison. All values were log-transformed prior to analysis. B) and C) Cell-cell fusion activity of KSHV gB is modestly activated by low pH. B) SLK target cells or C) Raji-EphA2 target cells were incubated with HEK293T effector cells that had been transfected to express the indicated glycoproteins (KSHV gB pCAGGS construct and RRV gB pcDNA6V5His constructs were used). pH was adjusted as indicated for 1h and then the cells were cocultured at normal pH. The experiments were performed three times in triplicates. Error bars represent the standard deviation. ns non-significant; * p<0.05; *** p<0.001, **** p<0.0001; two-way ANOVA with Dunnett multiple comparison test. All values were log-transformed prior to analysis.

Bafilomycin A1 is widely accepted as an agent that increases pH in endocytic vesicles. Nevertheless, we also sought to test our hypothesis of low-pH-triggered fusion in a more direct manner. We therefore let KSHV bind to Bafilomycin A1-treated cells at 4°C and then changed the pH to acidic conditions by adding citrate buffer. Acidification clearly increased KSHV infection under conditions of vacuolar ATPase inhibition (Fig 6). Even though KSHV infection was not fully restored to the original level, the increase due to acidification was 2.6 log10 (~400fold), whereas the acidification-mediated increase in lentiviral pseudotype infection driven by VSV-G, a well-studied pH-dependent fusogen [55], was only 1.1 log10 (~12fold). RRV infection was slightly affected by pH in the absence of Bafilomycin A1, and it was markedly enhanced (2.0 log10, ~100fold) by acidification in the presence of Bafilomycin A1.

To explore the possibility that the previously observed low fusion activity of KSHV gB (Fig 2A, 2C, 2D and 2F) was due to non-acidic pH, we tested the effect of pH on cell-cell fusion mediated by KSHV gB. We applied two different protocols, using PEI transfection or the more potent GenJet transfection protocol. In contrast to infection, cell-cell fusion with SLK target cells by KSHV gB was not enhanced to meaningful levels at low pH using the PEI transfection agent, likely reflecting the low fusogenicity of KSHV gB, while RRV gB-mediated and VSV-G-mediated cell-cell fusion were significantly enhanced by low pH (S6 Fig). We repeated the assay with KSHV gB using GenJet transfection, which affords higher gB expression levels. Using this protocol, we observed a weak signal for fusion of KSHV gH/gL/gB expressing effector cells with SLK target cells at pH5.5, which even if reaching significance was barely distinguishable from background (Fig 6B). Finally, we tested EphA2-overexpressing Raji cells as target cells for fusion at low pH (Fig 6C). In this setting, we indeed observed that at pH5.5 the KSHV core fusion machinery exhibited activity that differed significantly from background by approximately fivefold. At pH5 there was no discernable activity. It is unclear at present whether this reflects a very small pH window for KSHV gB’s activity or an increasingly negative effect of the low pH conditions on cell viability that was visually confirmed and particularly obvious after high efficiency transfection. Taken together, while KSHV gH/gL/gB was fusion-inactive under most conditions, weak activity could be observed when high-level expression was combined with mildly acidic conditions.

## Discussion

Using two novel molecular tools–a dual-fluorescent KSHV and a hybrid RRV expressing KSHV gB–as well as classical cell-cell fusion assays we demonstrate that KSHV gB exhibits low fusogenicity and requires vesicle acidification for the execution of fusion of the viral membrane with cellular membranes. While the potency of inhibitors of vesicle acidification against KSHV infection was well known, we now show conclusively that the entry step that is affected is the fusion process. Our hybrid RRV expressing KSHV gB further demonstrates that, specifically, KSHV glycoprotein B dictates the use of an endocytotic entry route, while wt RRV gB allows infection through a pathway that is independent of vesicle acidification and is functional e.g. in Raji cells. RRV infects many cells by an endocytotic route and is to a large degree dependent on vesicle acidification [33,54]. On the other hand, Raji cells with or without exogenous EphB3 receptor expression were infected by RRV in a largely Bafilomycin A1-insensitive manner (S5B Fig). RRV infection of BJAB was, to a small extent (between 10 and 20% of total infection), resistant to Bafilomycin A1 in a previous study [33], and RRV glycoproteins exhibit considerable cell-cell fusion activity. Thus, in contrast to KSHV, the preferred route of infection for RRV may be dictated by the target cell, which bears similarity to EBV infection except for the pH-dependence of RRV under many circumstances [22]. The presence of cellular restriction factors, such as e.g. the IFITM proteins or similar factors, might conceivably force these viruses into using one route or the other [42].

Overall, our data confirms that herpesviruses as a family can use infection via a low-pH route as a means of gaining access to cells. What is new in that respect is that KSHV exclusively relies on pH-dependent infection at least where tested so far, coinciding with strongly reduced fusion activity of its glycoproteins, specifically gB, at neutral pH, which sets it apart even from the closely related RRV.

The robust fusion activity of RRV’s core fusion machinery (Figs 2A, 2C, 2D, 2F, 6B and 6C) at neutral pH together with the clear increase in RRV gB-mediated fusion (Figs 6B, 6C and S6) and in RRV infection (Fig 6A) upon acidification are quite remarkable–RRV has seemingly evolved to master both routes of infection, endocytotic and direct membrane fusion, and may even spread through cell-cell fusion at neutral pH. On a speculative note, this generalist approach might ensure high infectivity under different conditions and is consistent with the high seroprevalence of RRV in macaques [56], while the reverse might be true for KSHV, whose seroprevalence is low outside endemic areas [2].

The literature is split between studies reporting robust or detectable fusion activity at least with C-terminally truncated KSHV gB [15,40,47] and others reporting insufficient activity for reliably measuring cell-cell fusion[35,57]. Our results reconcile these reports. While gBs of other gamma-herpesviruses (RRV, EBV) exhibit robust fusion activity, the gB of KSHV gB is a poor fusogen, whose activity is, at most, just above the background values (Figs 2 and 6), which is strongly influenced by the conditions and protocols used in the assays. Conflicting findings can be obtained by the use of different expression methods or assays, and activation of KSHV gB might occur more easily when cells are cultured for extended periods of time and the pH in the culture drops. Ultimately, we show that these experimental difficulties are rooted in the low intrinsic fusion activity of KSHV gB when directly compared to RRV gB at similar expression levels (Fig 2F).

Nevertheless, we were able to reliably demonstrate weak fusion activity of KSHV gB together with KSHV gH/gL at pH5.5 when we used a combination of optimized expression constructs and high-efficiency transfection (Fig 6B and 6C). This demonstrates that KSHV gB is activated by low pH, fully supporting our findings obtained in the dual-fluorescent virus system and with the KSHV gB hybrid RRV. In light of the robust fusion activity observed with RRV gB or VSV-G, our results do not, by any means, suggest that cell-cell fusion is naturally occuring at relevant levels during KSHV infection, even in e.g. a low-pH tissue compartment.

In the presence of an inhibitor of vesicle acidification, KSHV infection was more markedly reduced than RRV infection, and rescue of infection by lowering the pH exogenously was more pronounced. While we cannot rule out that there are certain conditions, that are not represented in our experimental systems and favor KSHV infection by direct fusion at the plasma membrane, at least for cell-free virus, this does not seem to be the norm according to our data and reports by others [14,16,17,19,20,33,44]. It should be noted that reaching a low pH environment on its own is likely not sufficient for efficient KSHV infection as KSHV gH/gL retains the ability to activate gB glycoproteins of other viruses very efficiently upon receptor binding, indicating a role of this glycoprotein complex in controlling the fusion process through receptor binding; in line with the established model of the herpesviral core fusion machinery that postulates binding of a proteinaceous receptor to activate fusion [10,35,43,57]. There may be additional determinants, cooperating with pH, that limit KSHV fusion activity mostly to endocytotic compartments, such as receptors specifically enriched in vesicles, low glycoprotein density or formation of higher-order glycoprotein complexes [58]. Our current working hypothesis is that KSHV gB has evolved to fuse efficiently from the viral but not a cellular membrane. Irrespective of the molecular underpinnings, our data using hybrid RRV expressing KSHV gB indicate that gB is the viral determinant that drives this specific mode of infection.

It is tempting to speculate why KSHV has evolved towards such low fusogenicity and pH dependence for fusion, in particular when contrasted with RRV gB. While RRV seems to have developed or retained the ability to fuse without the need for vesicle acidification at least in part, KSHV evolved towards an–at least where studied in detail–exclusively endocytotic entry pathway. One possible benefit of minimizing cell-cell fusion may be the reduction of tissue damage and the host immune response–a syncytial phenotype might be associated with increased pathogenicity and possibly also with increased genome instability and apoptosis of the host cell [59], a trait that may be selected against. Another conceivable benefit of fusion after rapid endocytosis may be the minimization of the display of viral glycoproteins on the cell surface, where they can be recognized by antibodies.

## Methods

### Ethics statement

Ethical approval was not sought for the present study because it did not involve human subject research or animal experiments. Immunization of mice and isolation of monoclonal antibody producing cells were performed by Genscript.

### Cell culture

Human embryonic kidney (HEK) 293T cells (ATCC) were kindly provided by the laboratory of Stefan Pöhlmann, German Primate Center—Leibniz Institute for Primate Research, Göttingen, Germany. SLK [60] cells (RRID:CVCL_9569) were originally obtained from the NIH AIDS Research and Reference Reagent program. Rhesus monkey fibroblasts (RF) were kindly provided by the laboratory of Rüdiger Behr, German Primate Center—Leibniz Institute for Primate Research, Göttingen, Germany. If not stated otherwise, all cells were cultured in Dulbecco’s Modified Eagle Medium (DMEM), high glucose, GlutaMAX, 25mM HEPES (Thermo Fisher Scientific) supplemented with 10% fetal bovine serum (FBS) (Serana Europe GmbH), and 50μg/ml gentamycin (PAN Biotech) (D10). iSLK cells [61] were maintained in D10 supplemented with 2.5μg/ml puromycin (InvivoGen) and 250μg/ml G418 (Carl Roth), for selection of iSLK carrying KSHV BACs 200μg/ml hygromycin was added. HUVEC (PromoCell, Heidelberg, Germany) were maintained in standard Endothelial Cell Growth Medium 2 (PromoCell, Heidelberg, Germany). Raji cells (RRID:CVCL_0511) (kindly provided by the laboratory of Jens Gruber), were cultured in RPMI (Thermo Fisher Scientific) supplemented with 10% FBS and 50μg/ml gentamycin (R10).

### Plasmids

The AX304 pLenti-CMV-BLAST-EphB3-Strep lentiviral expression plasmid encoding human EphB3 (NP_004434.2) was constructed by inserting the EphB3 open reading frame as described for pLenti-CMV-BLAST-EphA7-Strep, the EphA2 encoding AX283 was similarly constructed and encodes NP_004422.2 [37]. The mScarletH KanS shuttle plasmid containing the Kanamycin and i-SceI encoding recombination cassette for Red-mediated recombination was generated by inserting the EPKanS cassette after nucleotide 375 of mScarletH followed by a repeat (nucleotides 289 through 375) into pmScarlet-H_C1 (Addgene Plasmid #85043, a gift from Dorus Gadella [62]) using Gibson Assembly and the following PCR products: (i) with pmScarlet-H_C1 as template and oligos ScarletH_289:309_s (gtgatgaacttcgaggacgg) and SV40Promoter_as_KanR_OV (ctcgtcaagaaggcgatagaagcctaactgacacacattcc), (ii) with pmScarlet-H_C1 as template and oligos KanR_s (cttctatcgccttcttgacgag) and ScarletH_ 354:375_as

(gagcttcaccttgtagatcagg), and (iii) with pEPkan-S (Addgene Plasmid #41017, a gift from Nikolaus Osterrieder [63]) as template and oligos EPKanSfor_ScarletH354:375ov

(cctgatctacaaggtgaagctcGGATGACGACGATAAGTAGGGATAAC) and EPKanSrev_ScarletH289:309ov (ccgtcctcgaagttcatcacCAACCAATTAACCAATTCTGATTAG).

The AX185 mNeonGreen shuttle plasmid was also generated by Gibson Assembly. Using pNCS mNeonGreen (Allele Biotech) as a template a vector fragment was generated with primersmNeonGreen 463–482 for (TACCCCAACGACAAAACCAT) and mNeonGreen 504–523 rev (TGCCATTTCCAGTGGTGTAA) and joined with the pEPkan-s derived cassette using pEPkan-S as a template with primers EPKans forward mNeon 504–523 ov

(TTACACCACTGGAAATGGCAGGATGACGACGATAAGTAGGGATAAC) and EPKansS reverse mNeon 463–482 ov

(ATGGTTTTGTCGTTGGGGTACAACCAATTAACCAATTCTGATTAG).

### Lentiviral transduction

For production of lentiviral particles, semi-confluent HEK 293T cells in 10cm cell culture grade petri dishes were transfected with 1.4μg pMD2.G (VSV-G envelope expressing plasmid, a gift from Didier Trono (Addgene plasmid #12259), 3.6μg psPAX2 (Gag-Pol expression construct, a gift from Didier Trono (Addgene plasmid #12260), and 5μg of lentiviral expression constructs using PEI as described before [37]. The supernatant containing the pseudotyped lentiviral particles was harvested 2 to 3 days after transfection and filtered through 0.45μm CA membranes (Millipore). For transduction, lentivirus stocks were used at a 1:5 dilution unless stated otherwise. After 48h, the selection antibiotic blasticidin (Invivogen) was added to a final concentration of 10μg/ml. After initial selection the blasticidin concentration was reduced to 5μg/ml or removed (fusion assay) depending on the experiment.

### Recombinant viruses

Recombinant dual fluorescent viruses were based on BAC16 with the GFP open reading frame replaced with a Zeocin resistance gene and on this BAC carrying a mNeonGreen-GS-ORF65 fusion protein as described earlier, here abbreviated BAC16 mN65 [42]. Using a two-step, markerless λ-red-mediated BAC recombination strategy as described by Tischer et al. [63], mNeonGreen was replaced with the mScarletH open reading frame to generate BAC16 mS65. The mNeonGreen or mScarletH open reading frames were then C-terminally fused to the ORF39 open reading frame, generating BAC16 mS65-39mN and BAC16 mN65-39mS using Primers Bac16_ORF39-GSA-mScarletHmNEON_s

ATGAAAGTGACAGTGAAATCGACGAAACGCAAATGATATTCATTGGAAGCGCTGTGAGCAAGGGCGAGG and

Bac16_ORF39-GSA-mScarletHmNEONas

ATGGAGGAAGAGGGATGGGTTTATAATGCCAATATATCAGCTACTTGTACAGCTCGTCCATGCC to insert the respective fusion protein open reading frame containing the pEPkan-S selection/recombination cassette and a sequence repeat for insertion and scarless excision of the selection marker as described by Tischer et al. [63]. RRV-YFP (MN488839.2)[33,36] was modified using a recombination cassette generated from recombination template AX438—pcDNA6-KSHVgB-KanS_shuttleplasmid using primers “KSHV gB from aa6 on to RRV” TTTAAAGACCTGTACGCTCTTCTGTACCATCACCTGCAACTGTCCGACGGCCATGATGATAACTAACagattggccaccctgggg and „KSHV gB to RRV rev”GGGTGCGCGAATCGATTGGCCGCGCGGCTCTGGCGGGCGGCAAGTACAGGCGTGTGGGTGTGGTTActcccccgtttccggactg to generate RRV-YFP_KgB, replacing the orf8 gene with that of KSHV. In order to preserve the overlapping RRV ORF7, the first five amino acids of KSHV gB were replaced with those of RRV gB, which according to SignalP prediction does not alter signal peptide functionality [64]. AX438 encodes KSHV gB and the recombination cassette of pEPkan-S [63] after nucleotide 1560, followed by a 87 nucleotide repeat (nucleotides 1478–1560). Sequences of all viruses were confirmed by Illumina-based next generation sequencing. Reads have been deposited at BioProject, accession PRJNA1132903. Expression of RRV gB from RRV-YFP was confirmed by Western blot analysis as described previously [36] using anti-RRV gB monoclonal antibody 3H8.1 (laboratory of Scott Wong), expression of KSHV gB from RRY-YFP_KgB was confirmed using KSHV ORF8 rabbit polyclonal antibody (PAB14292, Abnova) and HRP-coupled anti-rabbit secondary antibody. GAPDH was detected as a loading control using mouse monoclonal antibody at 1:50000 (clone 1E6D9, proteintech) and HRP-coupled anti-mouse secondary antibody.

### Production of anti-KSHV gB antibody 7C2 and KSHV gB immunogen

Monoclonal antibody 7C2 was produced by a hybridoma cell line obtained from mice that were immunized with recombinant and purified KSHV gB ectodomains. The gB ectodomain expression construct included the synthetic KSHV gB gene (GeneScript) encoding residues 27–686 (UniProt access code F5HB81), which was codon optimized for expression in mammalian cells and cloned into the pCAGGs vector [48], along with the exogenous human IgG2 signal peptide (MGWSCIILFLVATATGVHS) at the 5’ end and an enterokinase cleavage site, followed by a Twin Strep-tag® (IBA Biosciences) at the 3’ end (DDDDKGGRSG WSHPQFEKGGGSGGGSGGSSAWSHPQFEK). The fusion loop residues were mutated to prevent protein aggregation. The protein was expressed in Expi293 cells (Thermo Fisher Scientific) according to the manufacturer’s protocol. The supernatants were collected five days after transfection, and the protein was affinity purified on a 5 ml Streptactin column (IBA Biosciences), followed by size exclusion chromatography on a Superdex S200 column (Cytiva). To avoid eliciting unwanted immune responses, the Twin Strep-tag was removed by enterokinase (New England Biolabs) digestion. Six units of enterokinase were used per 1 mg of gB in the volume where the final gB concentration was 1.7 mg/ml. The incubation was performed overnight at 25°C, and the mixture was run over a 0.2 ml gravity streptactin column (IBA Biosciences) to remove uncleaved material. The flow-through, containing the gB without the tag, was concentrated and further purified on a Superdex S200 size exclusion column using PBS as the running buffer. Aliquots of the purified gB were flash-frozen in liquid nitrogen and sent to GenScript, where mice were immunized and hybridoma cells were obtained using standard protocols [65].

### Infection assays, immune fluorescence and flow cytometry

The Raji cells in suspension were directly infected with the indicated amount of virus after being seeded in 96-well plates (25,000 cells/well). At 48 hpi suspension cells were centrifuged briefly, the supernatant was discarded and the cells were re-suspended in PBS. Then, the cell suspension was transferred to an equal volume of PBS supplemented with 4% methanol-free formaldehyde (Carl Roth) for fixation. For assessing Bafilomycin A1 inhibitory activity on Raji infection, the cells were plated at 96-well plate, 20,000 cells/well in 50 μL. Then, 50 μL Bafilomycin A1 at twice the indicated concentration was added to cells; the final concentration given in the figure was the one used during incubation. After 30 min incubation, the relevant virus was added to the cells. The virus volume was 25 μL, so the concentration of Bafilomycin A1 did not change by more than 25% between pre-incubation and post-infection. At 48 h post-infection, cells were harvested and analyzed by flow cytometry to determine the percentage of YFP-positive cells.

For all infection assays a minimum of 5,000 cells were analyzed per well for YFP expression on an ID7000 spectral analyzer (Sony).

For analysis of cell surface expression, transfected HEK293T cells were harvested one or two days post transfection, fixed with 4% methanol-free formaldehyde (Carl Roth) for 5 min followed by two washes in PBS. Non-specific binding sites were blocked for at least 30min in 10% FBS in PBS. The cells were stained in flow cytometry tubes or 96-well-plates in 50μl with 7C2 hybridoma or control supernatant at 1:50 (one experiment) or 1:100 (two experiments) dilution or with anti-Strep antibody (1:100, anti-Strep-Tag Classic, BioRad MCA2489) or control isotype (Invitrogen) at the same concentration in 10% FBS in PBS for 90min, followed by one wash with approx. 2ml (tube) or 200μl PBS (96-well-plate), followed by incubation with secondary antibody to mouse IgG coupled to AlexaFluor 647 (1:500, Thermo) 10% FBS in PBS for 1h. The cells were then washed once with approx. 2ml (tube) or 200μl (96-well-plate) PBS and post-fixated in 100μl 4% methanol-free formaldehyde until analysis of at least 5,000 cells on an ID7000 spectral analyzer (Sony).

For immunofluorescence, cells were seeded at approx. 75 000 cells (half that number for HFF) per well on 12-mm coverslips (YX03.1; Carl Roth) in 24-well plates. For experiments using inhibitors, incubation with virus suspension was performed in the presence of inhibitors or solvent controls at the indicated concentrations and medium was exchanged to D10 containing inhibitors or the respective solvent controls. On day two, the plate was cooled down on ice, followed by inoculation with 500 or 1000μl cold virus suspension per well and 30min centrifugation at 4122g and 4°C, followed by another 10min at 4°C. Then, the cells were washed three times in ice-cold PBS, followed either by fixation or medium exchange to D10 and incubation for the indicated timepoints at 37°C in a cell culture incubator, followed by one wash in PBS and fixation. The cells were fixed in 4% methanol-free formaldehyde in PBS for 10 min.

For the infection assay involving acidification, the cells were first cooled down and infected on ice with cold virus for 30min by spin-infection at 4200rpm, followed by a further 10min at 4°C and two washes with cold PBS. Then the medium was changed to 500μl cold D10 with or without citric acid on ice (pH was adjusted by addition of citric acid from a 1M stock and then the media were sterile filtered) and with 50nM bafilomycin A1 or the respective volume of DMSO solvent (control). The cells were then shifted to 37°C and incubated for 30min, followed by one wash with PBS and further incubation in D10 with 50nM bafilomycin A1 or the respective volume of DMSO solvent (control). The cells were harvested for flow cytometry after 24h as described above.

### Fluorescence microscopy and image analysis

Immunofluorescence was performed as described previously [42]. Z-stacks were imaged using ZEN software (Zeiss) and a laser scanning microscope (Zeiss SLM 800), frame size 2048px x 2048px, scan speed 5, pinhole was set using the 1AU setting provided by the Zeiss software, interval 0.31μm, one separate scan for the mScarletH channel, one for the mNeonGreen channel, and one for the Hoechst channel. For each image, first green positive regions of interest (ROI)s were defined and then red capsids were quantitated using the “find maxima” function of ImageJ in the whole image and in the green (ORF39-mNeonGreen)-positive ROIs.

For analysis of Fig 3, thresholding was run for mScarletH and mNeonGreen channels using uninfected cells as controls. The Colocalization Plugin (Pierre Bourdoncle) was used for identification of colocalized pixels in each image of the captured stacks. Generated single 8-bit images within the stacks were projected to one image using “Z project, max intensity” and colocalized clusters of pixels representing viral particles were counted using the “Find Maxima” function implemented in Fiji/ImageJ. Counting of single positive particles was performed using the same function on images after thresholding.

For analysis of Fig 4, colocalized regions in maximum projections from three independent stacks were identified as ROI using the “Colocalization Finder” Plugin by Philippe Carl. This technique was chosen as only minimal or no overlap of particles was observed in different z-planes. Limits for the ROIs were set so that no colocalization was observed in controls without virus or for single-fluorescent virus. The ROI containing the colocalized signals was then copied to the original image, and viral particles within this ROI were quantified using the “find maxima” function of ImageJ in the green channel image (in case of mNeonGreen-ORF65/ORF39-mScarletH particles), the same was also done for the whole image. The ratio of colocalized capsids to total capsids was then calculated for each image and averaged.

Live cells in culture were imaged on a Zeiss AxioVert.A1 cell culture microscope with LED fluorescence imaging.

### Cell-cell fusion assay

Cell fusion assays were performed as described previously [42,43,66]. Raji target cells were stably transduced with lentiviral constructs encoding EphB3 [43] or EphA2 [36], respectively. After selection, transduced Raji cells were again transduced with a lentiviral construct encoding a Gal4 response element-driven TurboGFP-luciferase reporter for 48 h [42]. SLK target cells were generated in an analogous manner. 293T effector cells were seeded in 96-well plates at 30,000 cells/well. One day after, 293T effector cells were transfected with a plasmid, encoding the Gal4 DNA binding domain fused to the VP16 trans-activator (VP16-Gal4), and the indicated viral glycoprotein combinations or a pcDNA empty vector control (VP16-Gal4: 31.25 ng/well, RRV gH: 12.5 ng/well, RRV gL: 62.5 ng/well, RRV gB: 18.75 ng/well, KSHV gH: 12.5 ng/well; KSHV gL: 62.5 ng/well, KSHV gB: 18.75 ng/well, pcDNA only: 93.75 ng/well) using PEI as described before [45]. Alternatively, GenJet (SignaGen Laboratories) was used as a transfection reagent according to the manufacturer’s instructions. 5 h after transfection, the medium with the transfection mix was removed and replaced with 100 μL fresh D10. The target cells were counted, and 40,000 target cells were added to 293T effector cells in 100 μL fresh R10 (or D10 for SLK target cells). Triplicate wells were analyzed for all target-effector combinations. After 48 h, cells were washed once in PBS and lysed in 35 μL 1× Luciferase Cell culture lysis buffer (E1531, Promega) for 15 min at room temperature and centrifuged at maximum speed for 10 min at 4°C. A volume of 20 μL of each cell lysate was used to measure luciferase activity using the Beetle-Juice Luciferase Assay (PJK), according to the manufacturer’s instructions on a Biotek Synergy 2 plate reader. For assays involving acidification, the respective low-pH media were prepared as detailed for infection assays and the cells were incubated at low pH for 1h.

## Supporting information

S1 Data GraphPad Prism files containing the numerical data used to generate the graphs and original image files of the Western blot figures. (ZIP)

S1 Fig (A) Fusion activity of different KSHV and RRV gB variants co-expressed with RRV gB in 293T effector cells with EphA2-overexpressing Raji target cells. **** p<0.0001; ordinary one-way ANOVA, Dunnett test for multiple comparison. All values were log-transformed prior to analysis. (B) Expression of different KSHV and RRV gB variants in transfected 293T cells. The numbering corresponds to the legend in (A). The Western blot was detected with an anti-His-Tag antibody at 1:1000 dilution (mouse monoclonal, clone 4E3D10H2/E3, Invitrogen) and anti-mouse HRP-coupled secondary antibody. Arrows indicate gB-specific reactivities. Constructs 1–5 use pCAGGS as a vector backbone, construct 6 pcDNA6V5His. Plasmid MB148 (IgG2 signal peptide followed by N-terminal tandem Strep tag II (WSHPQFEK-GGGSGGGSGGSA-WSHPQFEK) fused via an Ser-Arg encoding XbaI linker to RRV gB amino acids 26–829 codon optimized) was synthesized/constructed by Genscript using accession AAC58686.1 as template and pCAGGS as backbone. The KSHV gB coding sequence in AX223 was directly cloned from RRV-YFP (accession MN488839.2) into the pcDNA6V5His backbone. Plasmids MB113 (IgG2 signal peptide followed by N-terminal tandem Strep tag II fused via an Ser-Arg encoding XbaI linker to KSHV gB codon optimized), MB137 (IgG2 signal peptide followed by N-terminal tandem Strep tag II WSHPQFEK-GGGSGGGSGGSA-WSHPQFEK fused via an Ser-Arg encoding XbaI linker to KSHV gB codon optimized amino acids 27–845, C-terminal His-tag), MB157 (KSHV gB, C-terminal His-tag) and MB158 (KSHV gB L791A L792A Y830A, C-terminal His-tag) were synthesized/constructed by Genscript (accession MK143395.1 as template) using pCAGGS as backbone. c.o. = codon optimized. (C) 293T cells were transfected with the indicated constructs and surface expression was determined using 7C2 anti-gB antibody (hybridoma supernatant) or a control hybridoma supernatant. The experiment was repeated three times. The gBΔ58 construct was obtained by PCR mutagenesis of plasmid MB157.
(PDF)

S2 Fig Cell-cell fusion assay using KSHV and RRV wt and chimeric gB constructs. (A) The indicated chimeric constructs were tested in a cell-cell-fusion assay using EphA2-overexpressing Raji cells as target cells. Only significant differences from the “empty vector” (no viral glycoproteins) effector cell control are shown. **** p<0.0001; ordinary one-way ANOVA, Dunnett test for multiple comparison. All values were log-transformed prior to analysis. (B) Expression of the indicated gB constructs as determined by transfection into 293T cells and Western blot analysis (performed as in S1 Fig, fusion assay lysates were not suitable for Western blot). (C) Expanded legend of the individual constructs. Domains were determined using TMHMM [67].
(PDF)

S3 Fig Colocalization of capsids with gM was analyzed as the fraction of capsids colocalizing with gM (left) or the fraction of gM-positive spots colocalizing with capsids. Data from 12 0h-timepoint slides was analyzed. Error bars represent the standard error of the mean.
(PDF)

S4 Fig Swapping the position of mNeonGreen and mScarletH has no effect on measured virus-cell fusion activity and sensitivity of measured fusion activity to Bafilomycin A1. The ORF65 capsid protein was tagged with mNeonGreen and gM with mScarletH, resulting in KSHVmN65-39mS, (reversed order compared to before) and infection of SLK cells was carried out in the presence of solvent control or 50 nM Bafilomycin A1. The ratio of gM-mScarletH colocalized mNeonGreen-ORF65 capsids over total mNeonGreen-ORF65 capsids was calculated. Error bars represent the standard deviation, n = 3. ns non-significant; * p<0.05; two-way ANOVA with Sidak’s correction for multiple comparison.
(PDF)

S5 Fig **A)** Raji cells recombinantly expressing EphB3. Raji cells were transduced with empty vector or EphB3 expression construct. After selection with blasticidin at 10μg/ml, the cells were subjected to Western blot analysis using anti-EphB3 monoclonal antibody (mouse, clone 7E5, Santa Cruz Biotechnology) followed by detection with HRP-coupled anti-mouse secondary antibody. B) Sensitivity of RRV infection of Raji cells to Bafilomycin A1. Empty vector transduced Raji cells were infected with RRV-YFP in the presence of Bafilomycin A1 at the indicated concentrations.
(PDF)

S6 Fig Cell-cell fusion by RRV gB is increased by low-pH while KSHV gB-mediated cell-cell fusion does not reach significant levels at low pH under conditions using the PEI transfection protocol. A cell-cell fusion assay of effector cells expressing the indicated glycoproteins with SLK target cells was performed and pH was lowered for 1h, then the medium was changed to normal pH medium. We did not lower pH to 4.5 for the cell-cell fusion assay as prolonged incubation, which is needed for that assay, at pH 4.5 visibly damaged the cells. Error bars represent the standard deviation. Comparisons to no viral glycoprotein control within groups and for the same viral glycoprotein between groups, only significant comparisons are shown; **** p<0.0001; the experiment was performed in triplicate, for KSHV gBΔ58 two independently cloned constructs were used and the results pooled; two-way ANOVA with Sidak’s correction for multiple comparison. Comparisons were made to the “no viral glycoprotein” control in each condition and between DMEM and acidic conditions for each glycoprotein. All values were log-transformed prior to analysis.
(PDF)
