# Supplementary figures and images for: Kaposi’s sarcoma-associated herpesvirus (KSHV) gB dictates a low-pH endocytotic entry pathway as revealed by a dual-fluorescent virus system and a rhesus monkey rhadinovirus expressing KSHV gB
Source: PLoS Pathog. 2025 Jan 16;21(1):e1012846. doi: 10.1371/journal.ppat.1012846 (PMC11801733; doi:10.1371/journal.ppat.1012846)

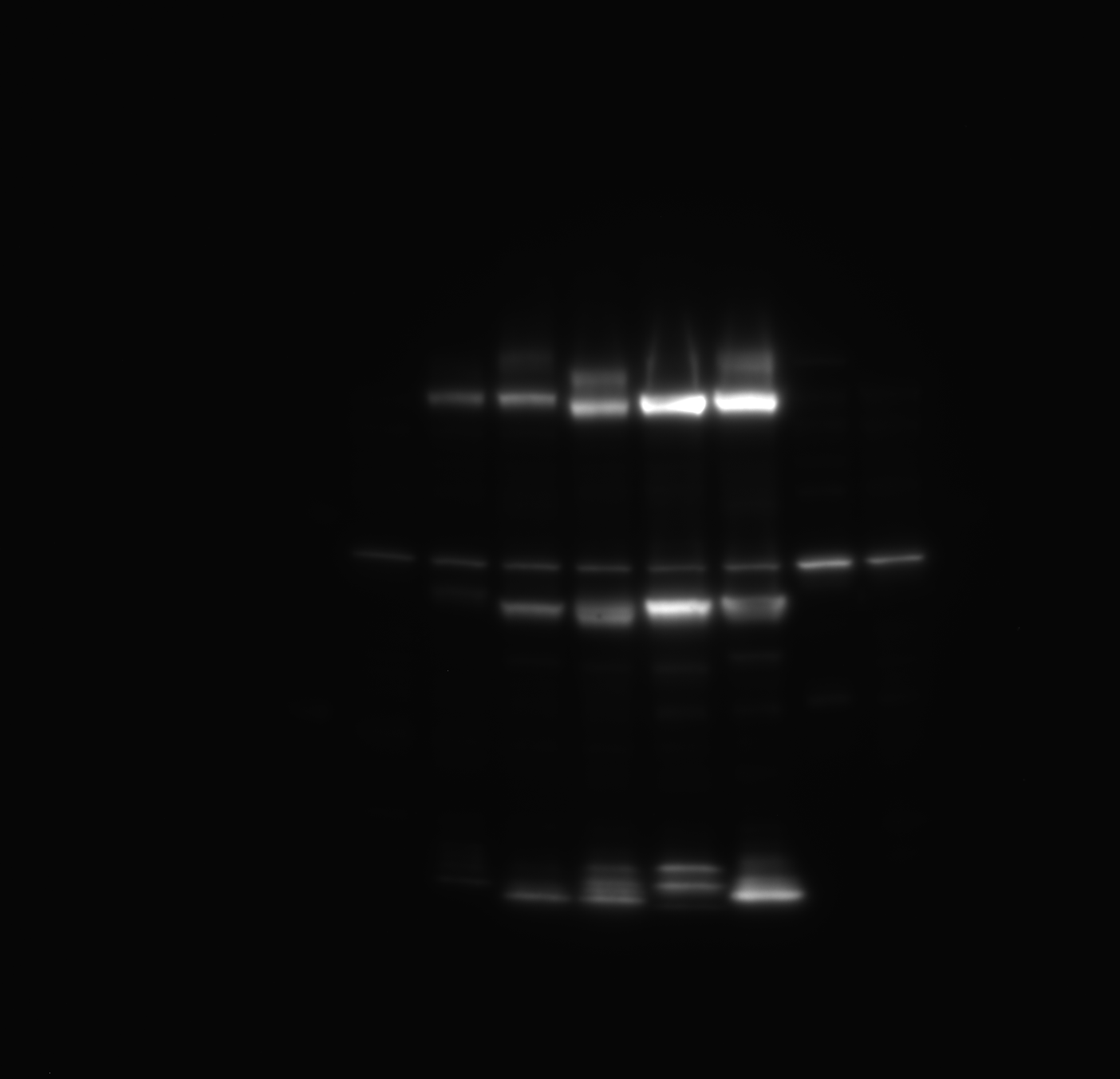

Supplement: S1 Data — (ZIP) [file ppat.1012846.s001.zip › Supplemental_Original_Data_Files/FigS1B_Western_Blot/293T_gB-constructs-expression_anti-His_lossless.tif]

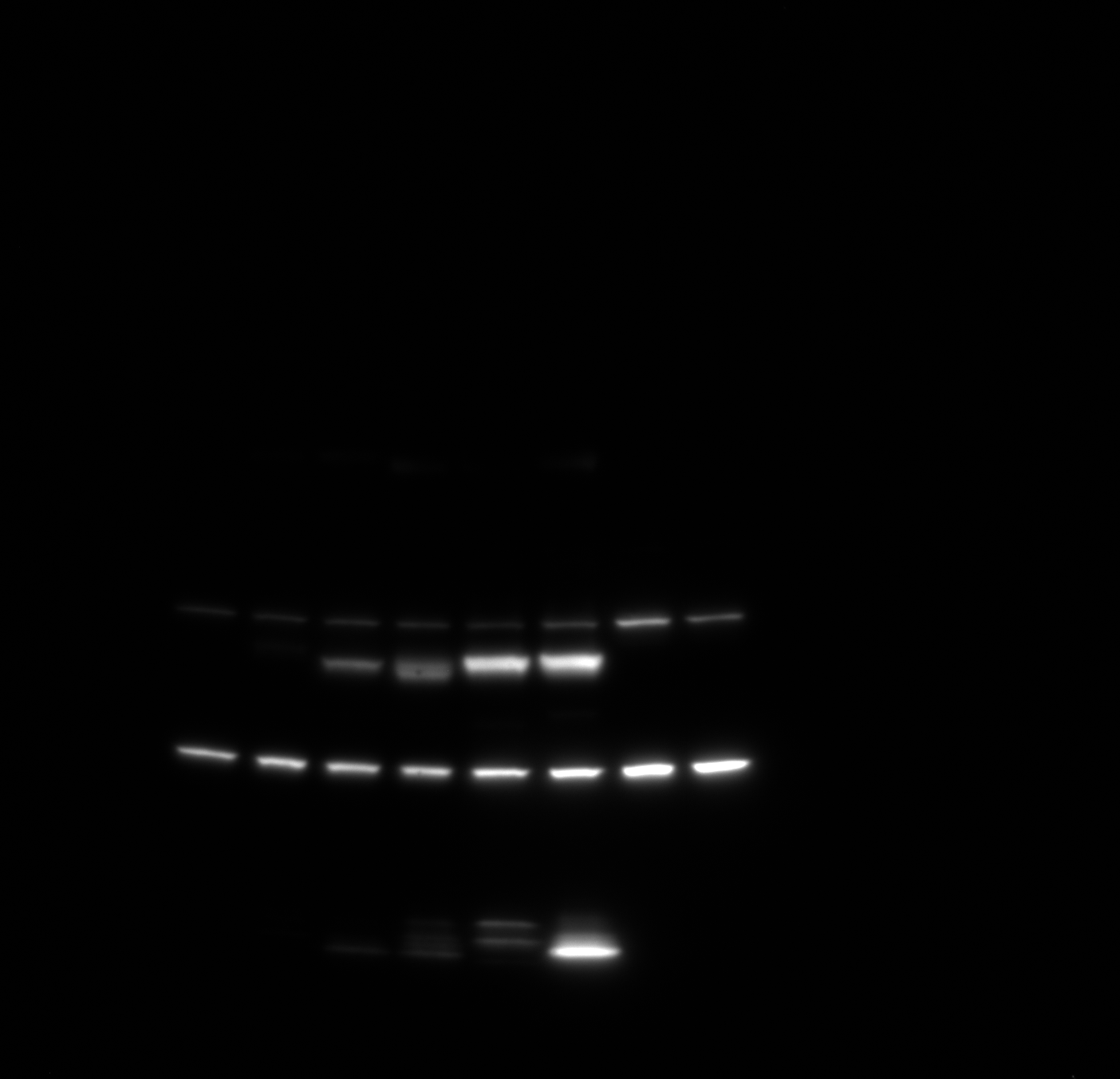

Supplement: S1 Data — (ZIP) [file ppat.1012846.s001.zip › Supplemental_Original_Data_Files/FigS1B_Western_Blot/293T_gB-constructs-expression_anti-GAPDH_lossless.tif]

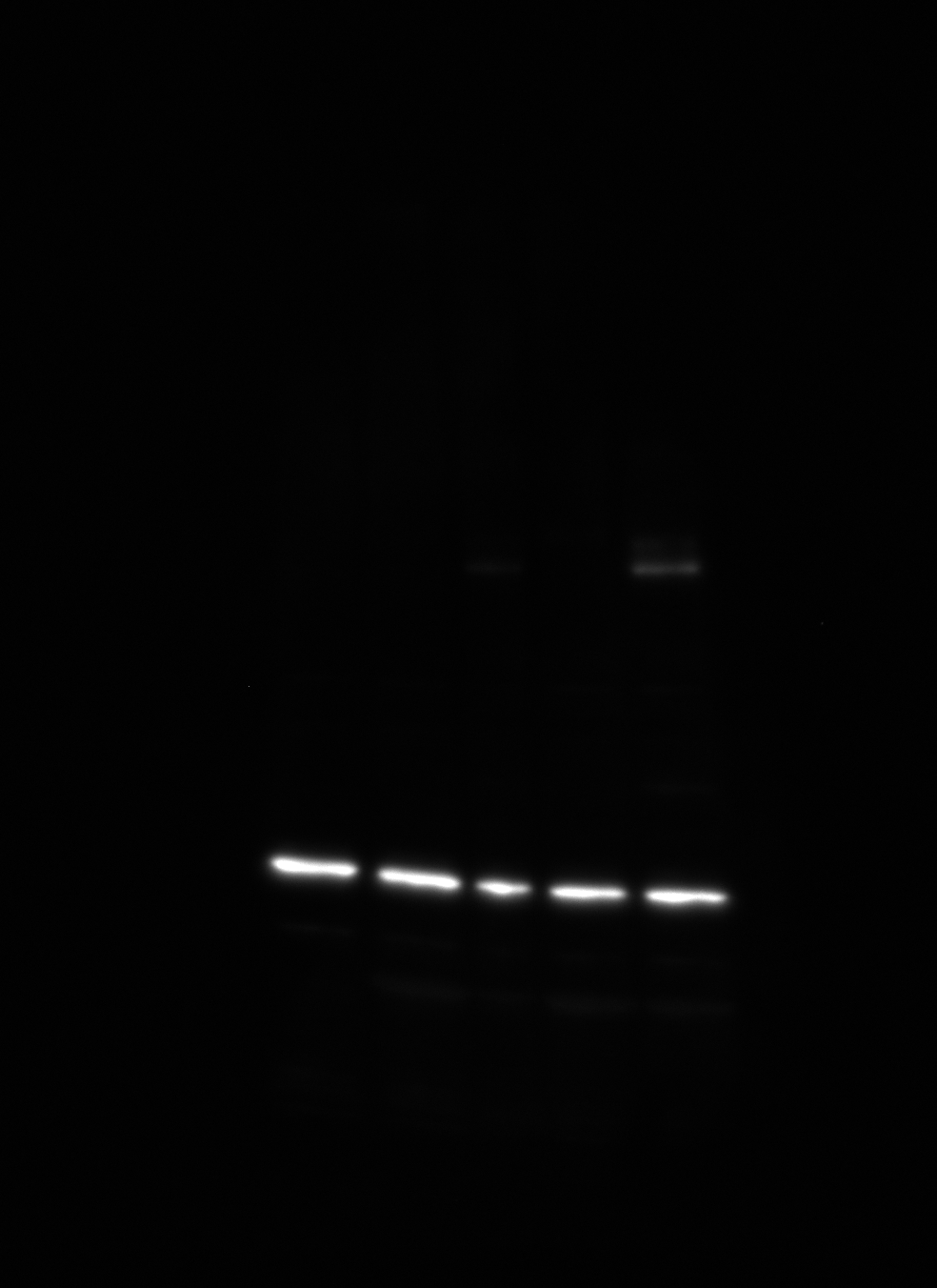

Supplement: S1 Data — (ZIP) [file ppat.1012846.s001.zip › Supplemental_Original_Data_Files/Fig5B_Western_Blot/Fig 5B WB-iSLK-RF-wt-kgB-anti-KSHVgB-anti-GAPDH_lossless.tif]

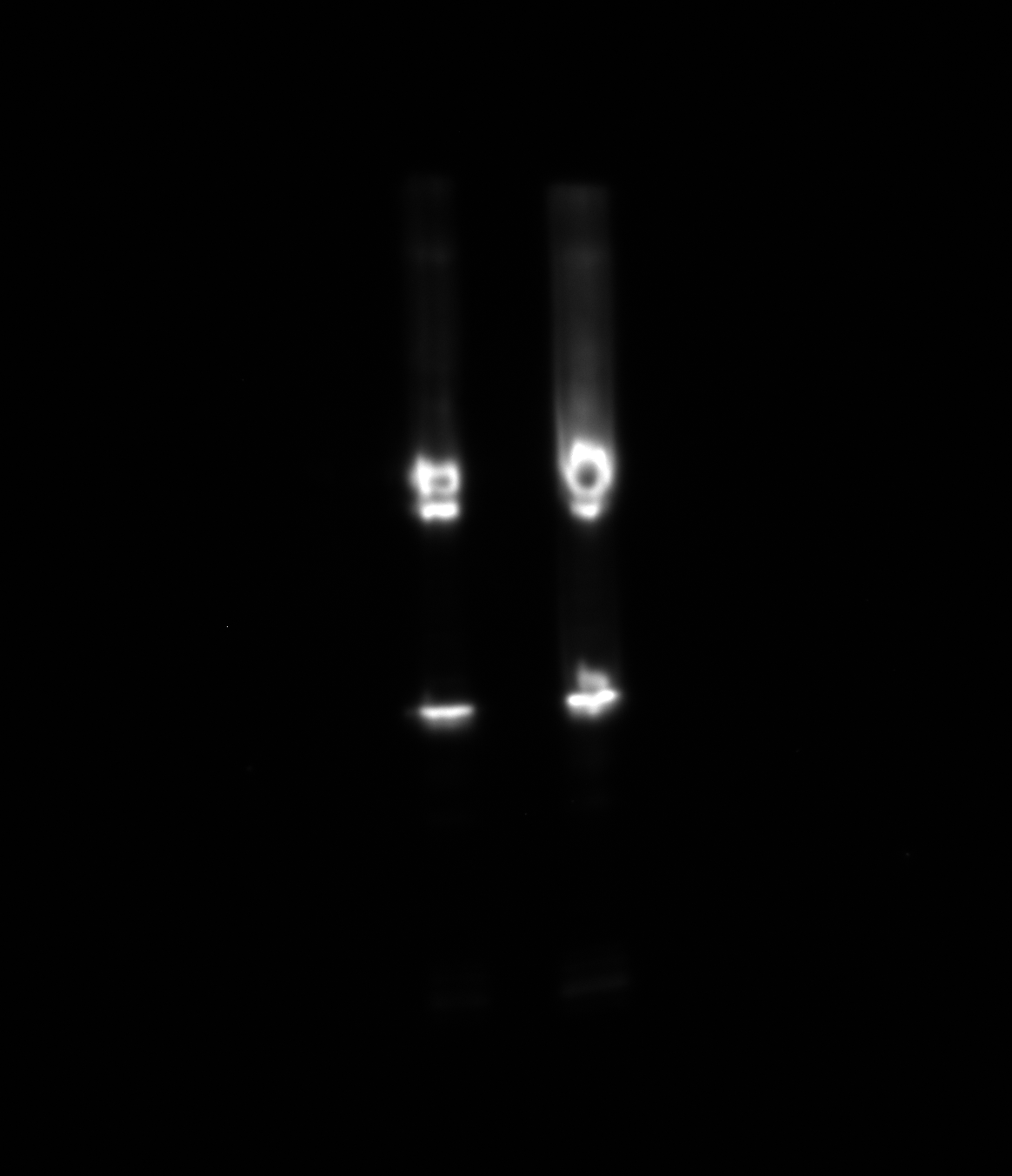

Supplement: S1 Data — (ZIP) [file ppat.1012846.s001.zip › Supplemental_Original_Data_Files/Fig5B_Western_Blot/Fig 5B WB-iSLK-RF-wt-kgB-anti-RRVgB_lossless.tif]

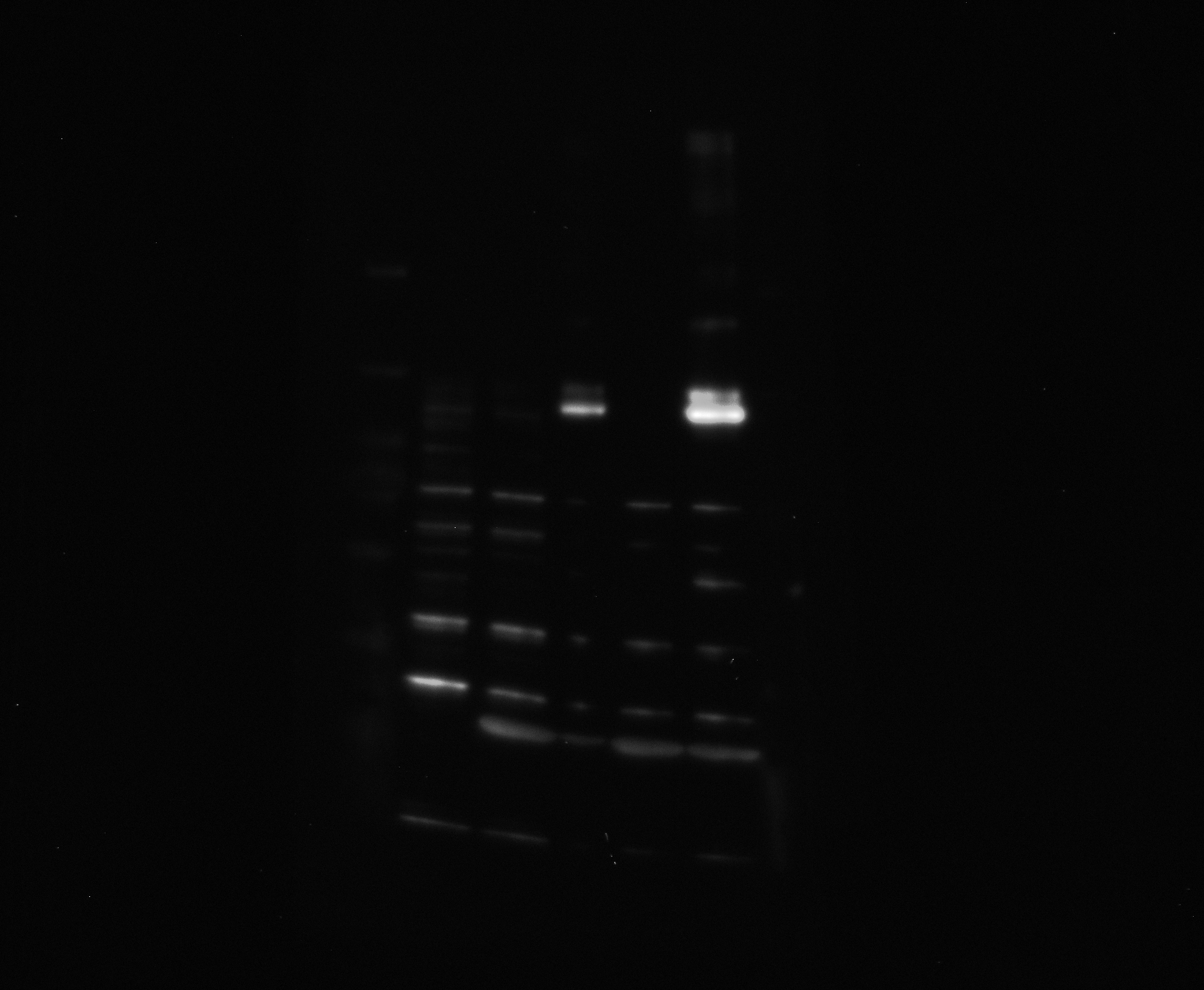

Supplement: S1 Data — (ZIP) [file ppat.1012846.s001.zip › Supplemental_Original_Data_Files/Fig5B_Western_Blot/Fig 5B WB-iSLK-RF-wt-kgB-anti-KSHVgB_lossless.tif]

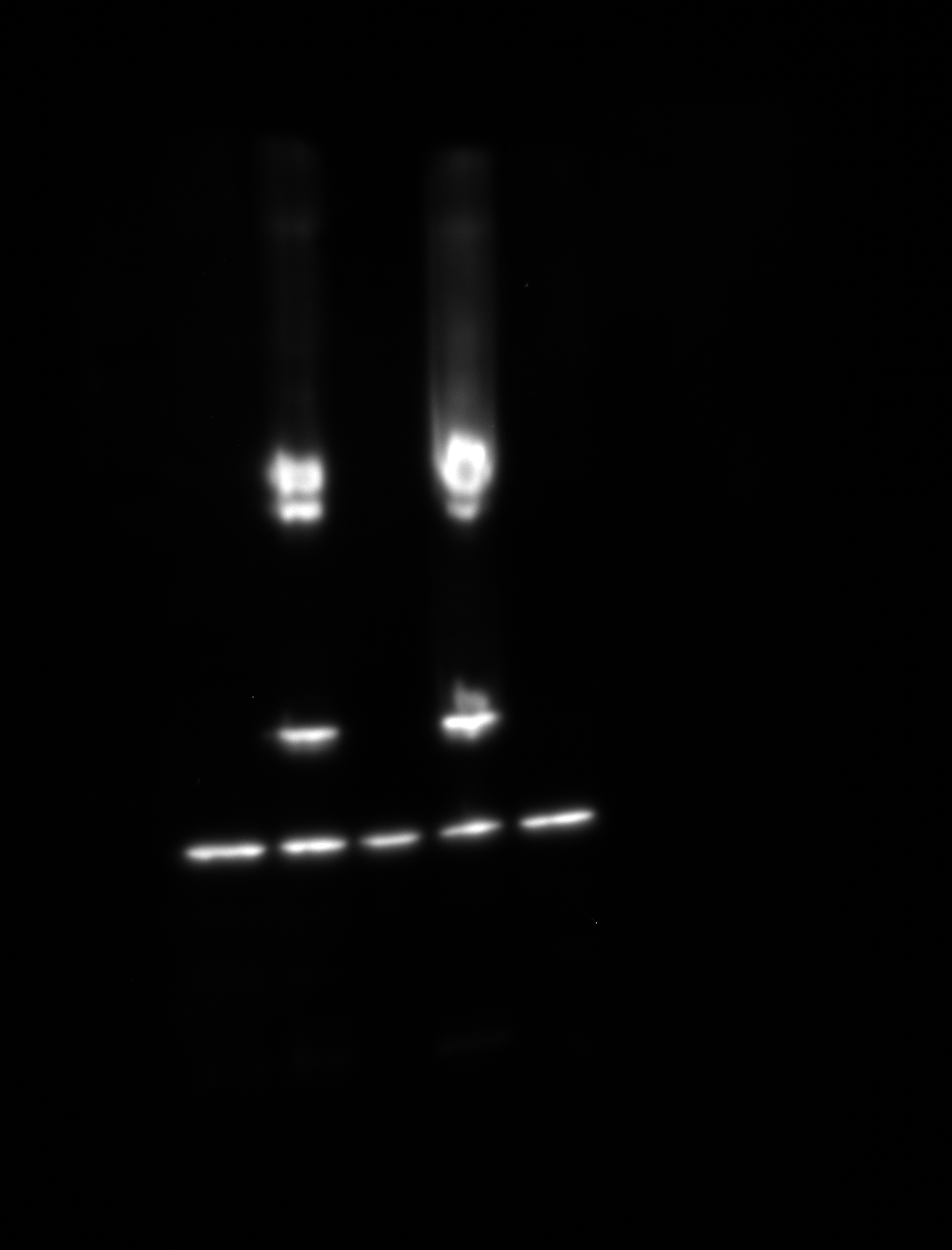

Supplement: S1 Data — (ZIP) [file ppat.1012846.s001.zip › Supplemental_Original_Data_Files/Fig5B_Western_Blot/Fig 5B WB-SLK-RF-wt-kgB-anti-RRVgB-anti-GAPDH_lossless.tif]

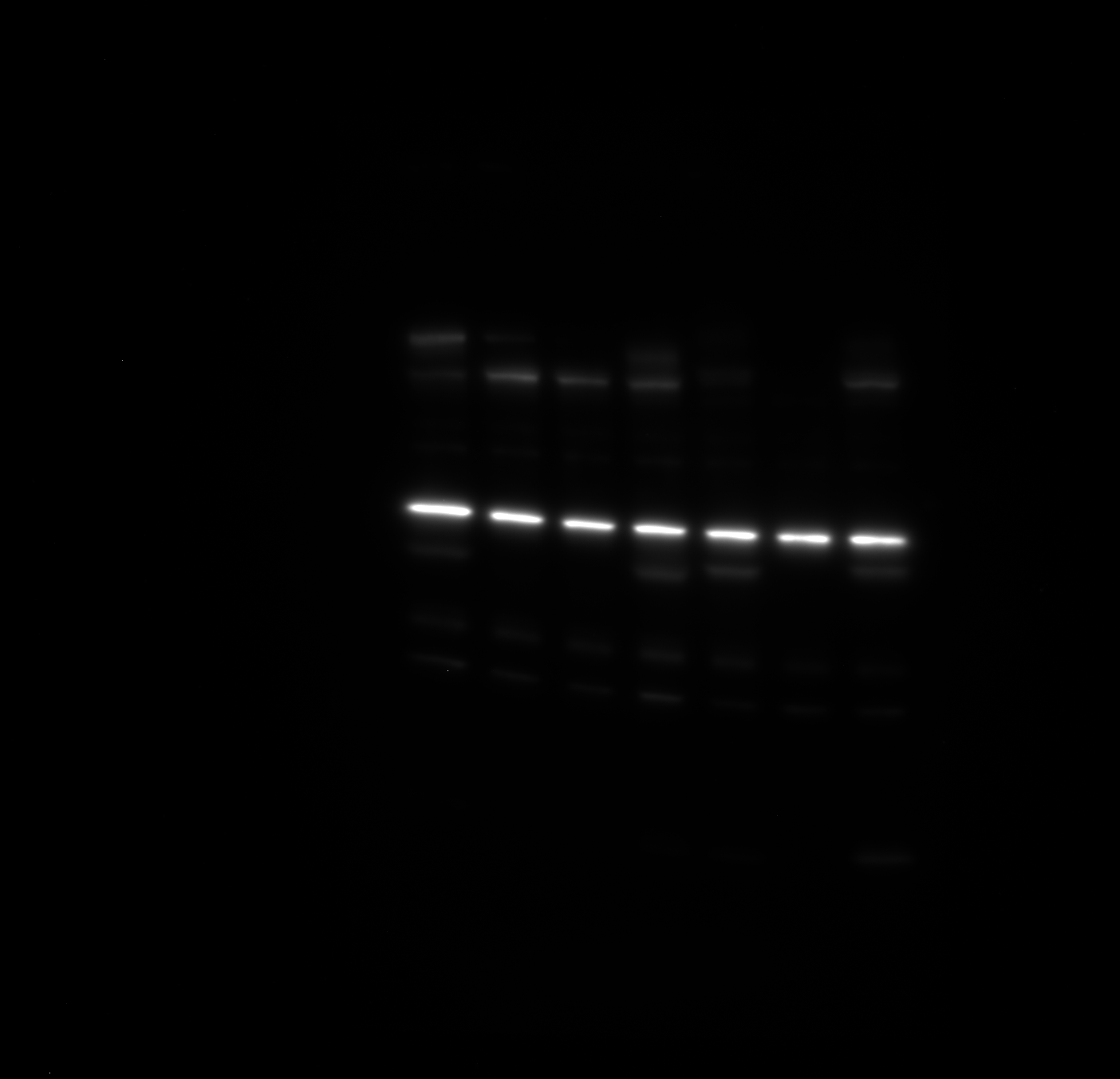

Supplement: S1 Data — (ZIP) [file ppat.1012846.s001.zip › Supplemental_Original_Data_Files/FigS2B_Western_Blot/SFig2B-anti-Hislossless.tif]

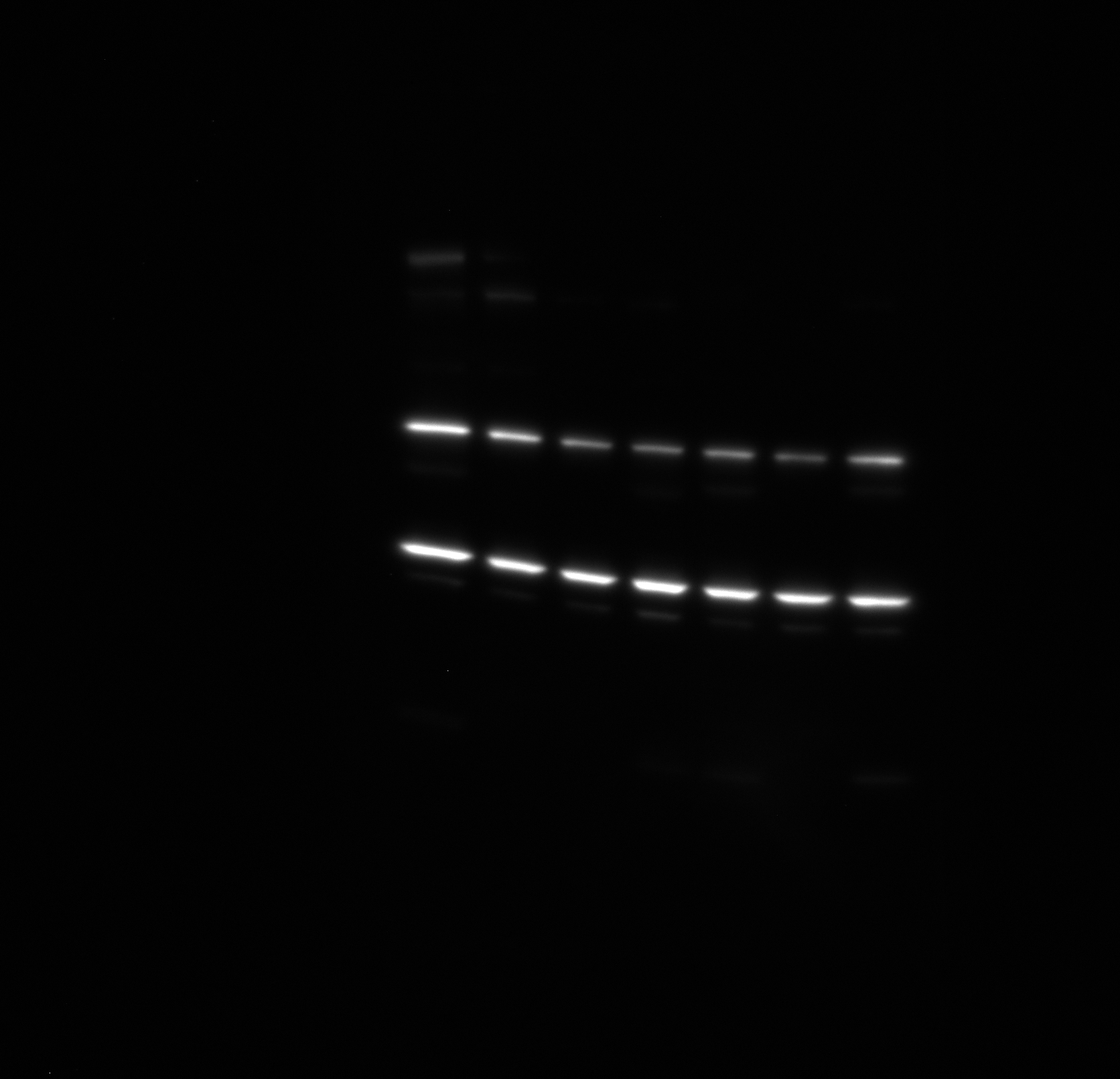

Supplement: S1 Data — (ZIP) [file ppat.1012846.s001.zip › Supplemental_Original_Data_Files/FigS2B_Western_Blot/SFig2B GAPDHlossless.tif]

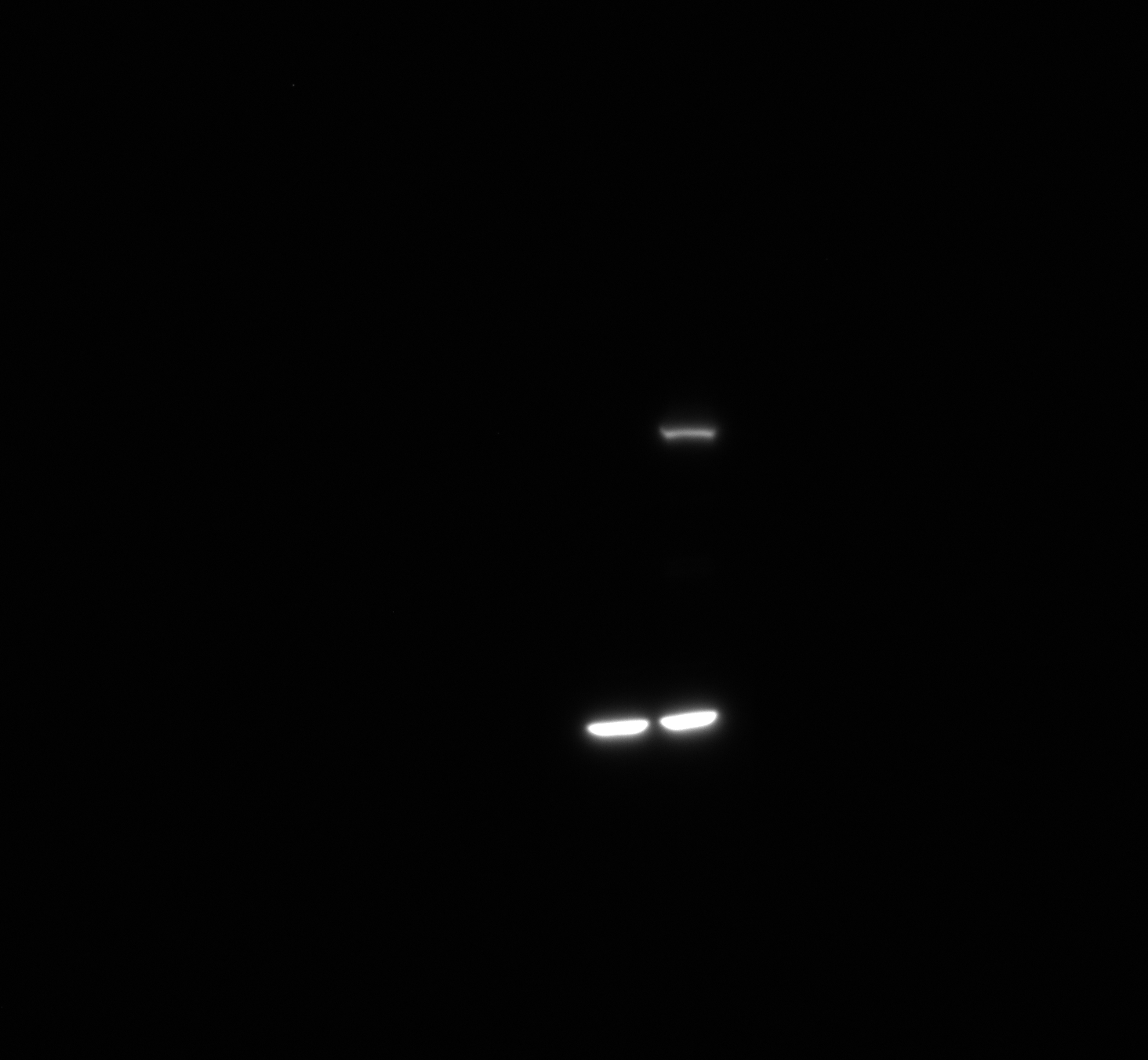

Supplement: S1 Data — (ZIP) [file ppat.1012846.s001.zip › Supplemental_Original_Data_Files/FigS5A_Western_Blot/Raji-transduced-anti-EphB3-anti-GAPDH_lossless.tif]

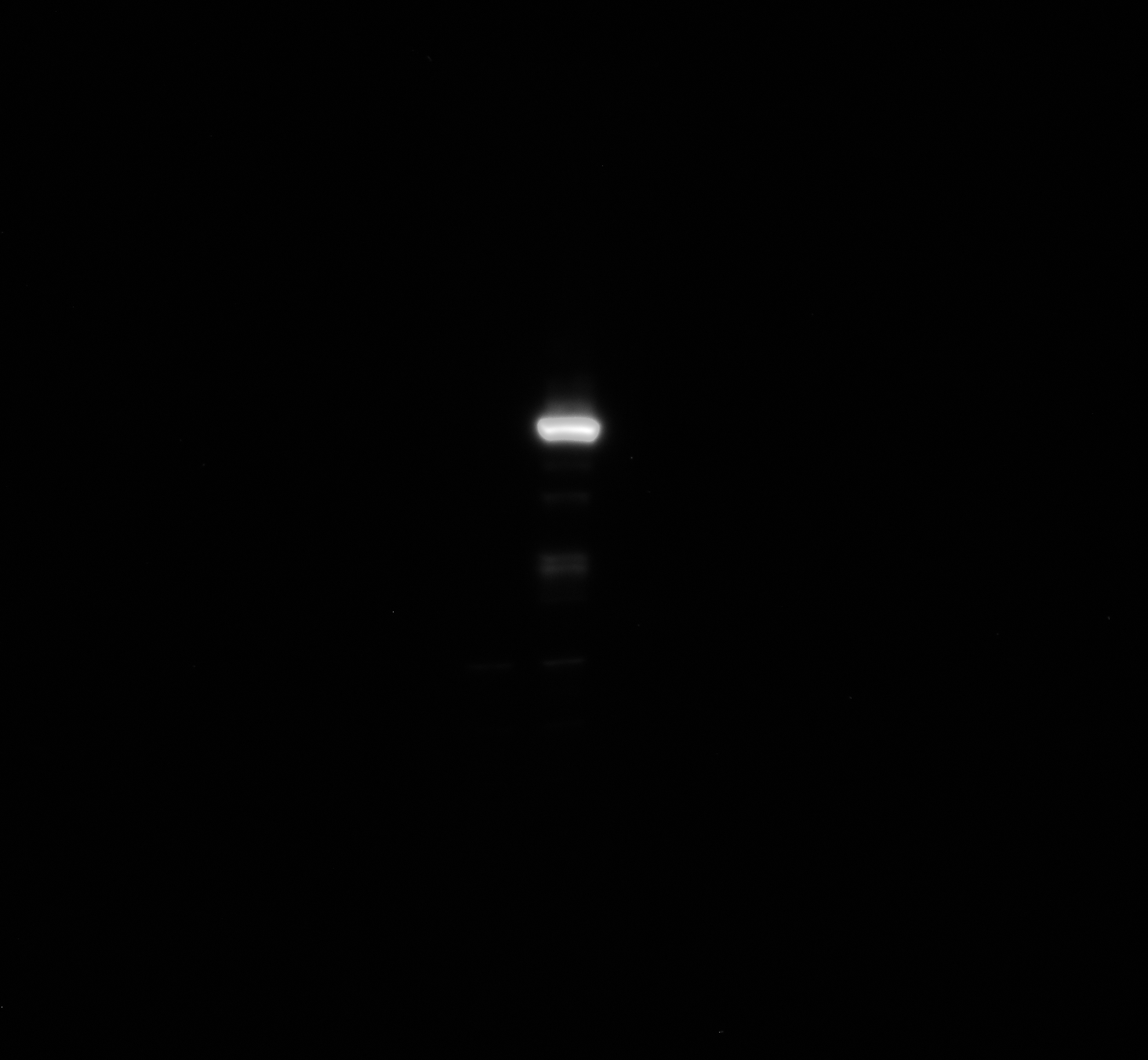

Supplement: S1 Data — (ZIP) [file ppat.1012846.s001.zip › Supplemental_Original_Data_Files/FigS5A_Western_Blot/Raji-transduced-anti-EphB3_lossless.tif]

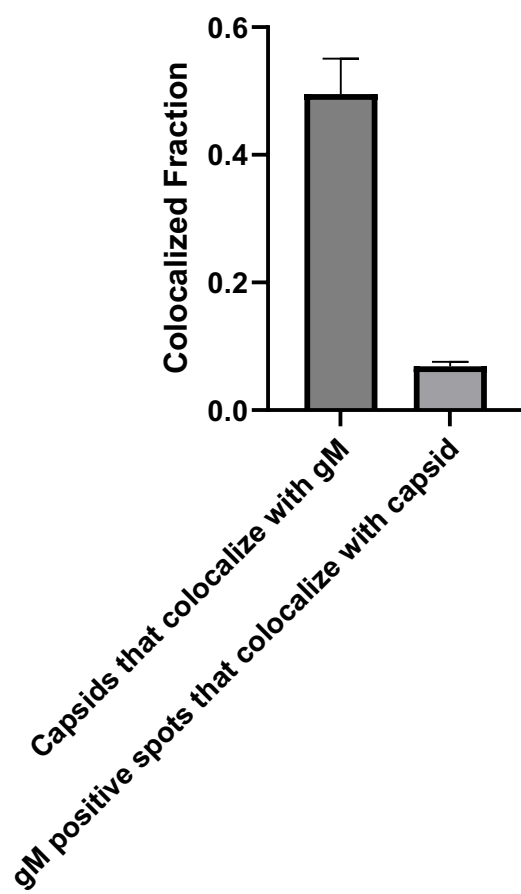

Supplement: S3 Fig — Data from 12 0h-timepoint slides was analyzed. Error bars represent the standard error of the mean. (PDF) [file ppat.1012846.s004.pdf]

Green capsid vs red glycoprotein gM

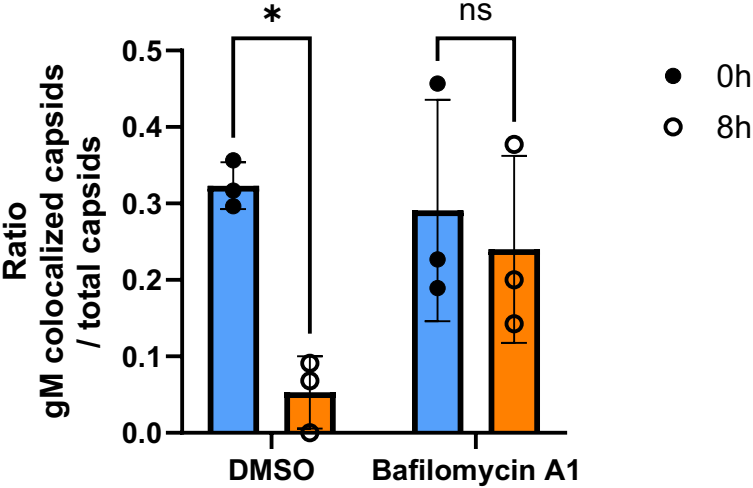

Supplement: S4 Fig — The ORF65 capsid protein was tagged with mNeonGreen and gM with mScarletH, resulting in KSHVmN65-39mS, (reversed order compared to before) and infection of SLK cells was carried out in the presence of solvent control or 50 nM Bafilomycin A1. The ratio of gM-mScarletH colocalized mNeonGreen-ORF65 capsids over total mNeonGreen-ORF65 capsids was calculated. Error bars represent the standard deviation, n = 3. ns non-significant; * p<0.05; two-way ANOVA with Sidak’s correction for multiple comparison. (PDF) [file ppat.1012846.s005.pdf]

A

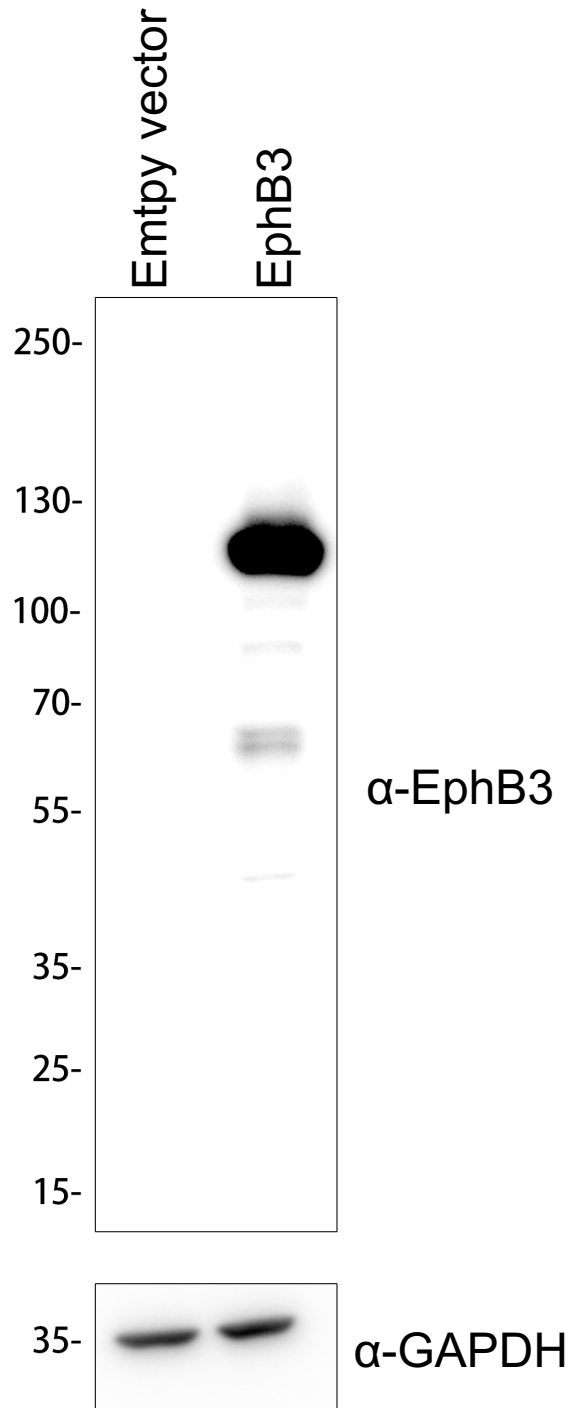

B

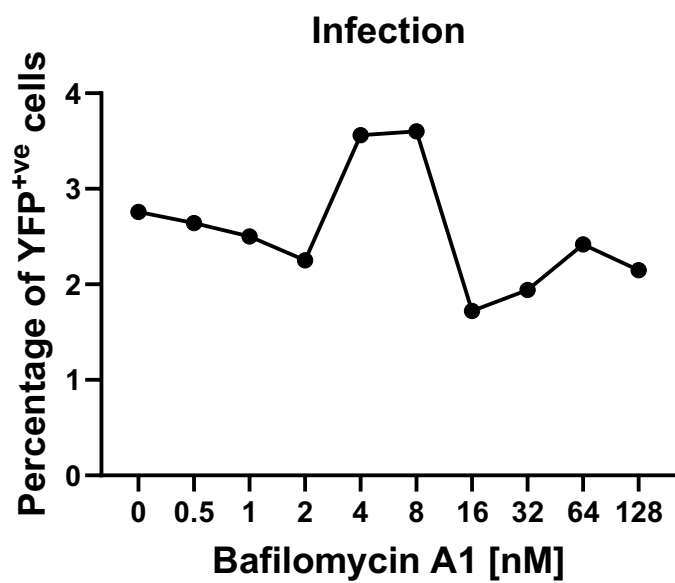

Supplement: S5 Fig — A) Raji cells recombinantly expressing EphB3. Raji cells were transduced with empty vector or EphB3 expression construct. After selection with blasticidin at 10μg/ml, the cells were subjected to Western blot analysis using anti-EphB3 monoclonal antibody (mouse, clone 7E5, Santa Cruz Biotechnology) followed by detection with HRP-coupled anti-mouse secondary antibody. B) Sensitivity of RRV infection of Raji cells to Bafilomycin A1. Empty vector transduced Raji cells were infected with RRV-YFP in the presence of Bafilomycin A1 at the indicated concentrations. (PDF) [file ppat.1012846.s006.pdf]

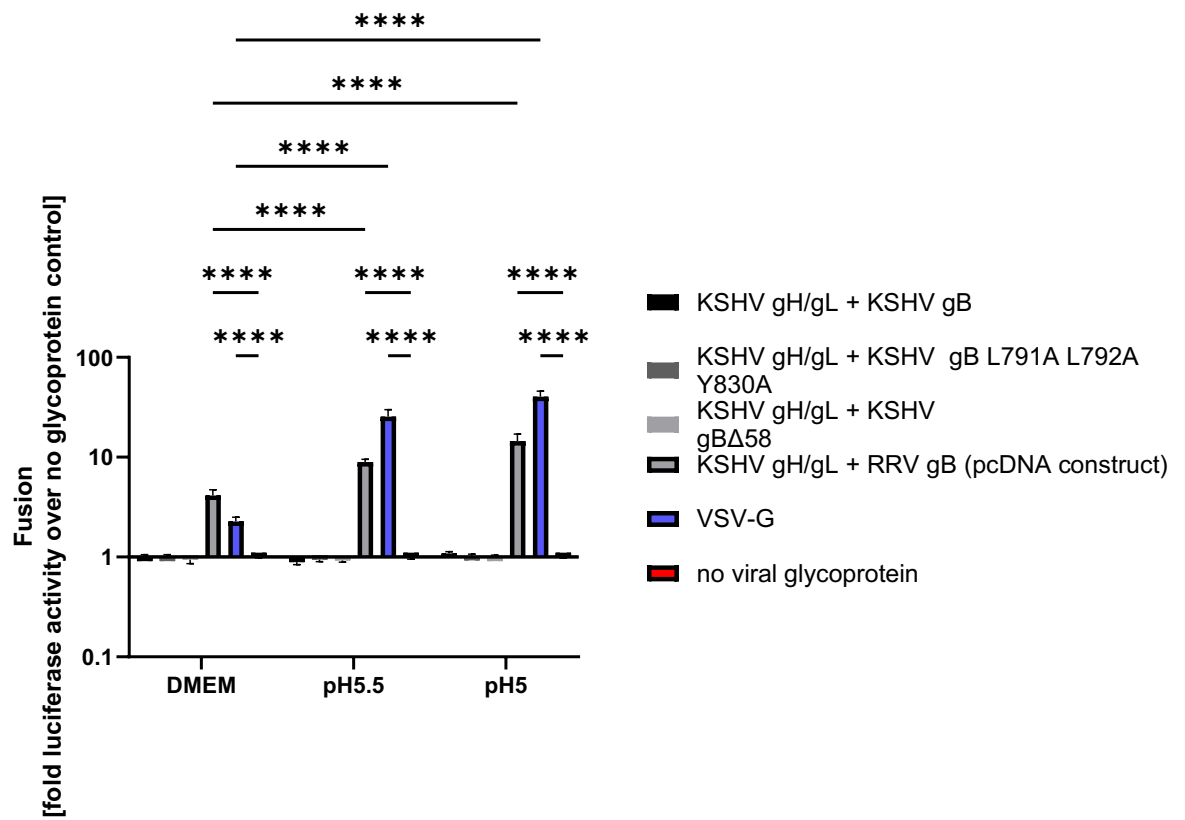

Supplement: S6 Fig — A cell-cell fusion assay of effector cells expressing the indicated glycoproteins with SLK target cells was performed and pH was lowered for 1h, then the medium was changed to normal pH medium. We did not lower pH to 4.5 for the cell-cell fusion assay as prolonged incubation, which is needed for that assay, at pH 4.5 visibly damaged the cells. Error bars represent the standard deviation. Comparisons to no viral glycoprotein control within groups and for the same viral glycoprotein between groups, only significant comparisons are shown; **** p<0.0001; the experiment was performed in triplicate, for KSHV gBΔ58 two independently cloned constructs were used and the results pooled; two-way ANOVA with Sidak’s correction for multiple comparison. Comparisons were made to the “no viral glycoprotein” control in each condition and between DMEM and acidic conditions for each glycoprotein. All values were log-transformed prior to analysis. (PDF) [file ppat.1012846.s007.pdf]
