# Supplementary material for: Kaposi’s sarcoma-associated herpesvirus (KSHV) gB dictates a low-pH endocytotic entry pathway as revealed by a dual-fluorescent virus system and a rhesus monkey rhadinovirus expressing KSHV gB
Source: PLoS Pathog. 2025 Jan 16;21(1):e1012846. doi: 10.1371/journal.ppat.1012846 (PMC11801733; doi:10.1371/journal.ppat.1012846)
Supplement: S2 Fig — (A) The indicated chimeric constructs were tested in a cell-cell-fusion assay using EphA2-overexpressing Raji cells as target cells. Only significant differences from the “empty vector” (no viral glycoproteins) effector cell control are shown. **** p<0.0001; ordinary one-way ANOVA, Dunnett test for multiple comparison. All values were log-transformed prior to analysis. (B) Expression of the indicated gB constructs as determined by transfection into 293T cells and Western blot analysis (performed as in S1 Fig, fusion assay lysates were not suitable for Western blot). (C) Expanded legend of the individual constructs. Domains were determined using TMHMM [67]. (PDF) [file ppat.1012846.s003.pdf]

A

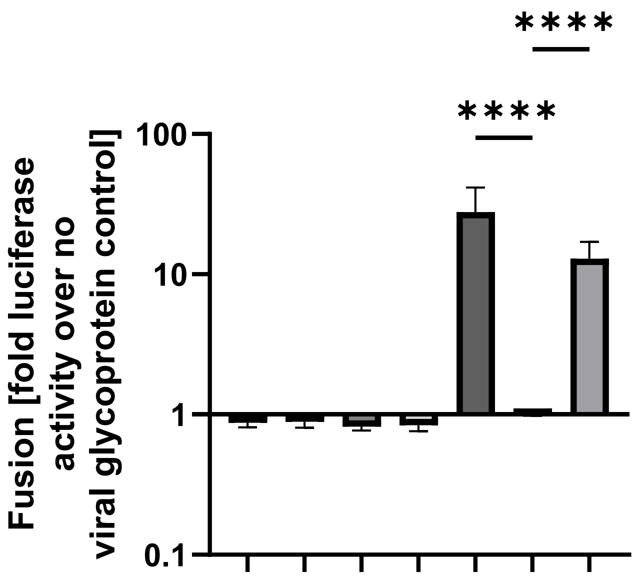

ECD	K	R	R	K	R	-	R
TM	R	K	R	K	R	-	R
ICD	R	K	K	K	R	-	R
KSHV gH/gL	+	+	+	+	+		+
#	1	2	3	4	5	6	7

B

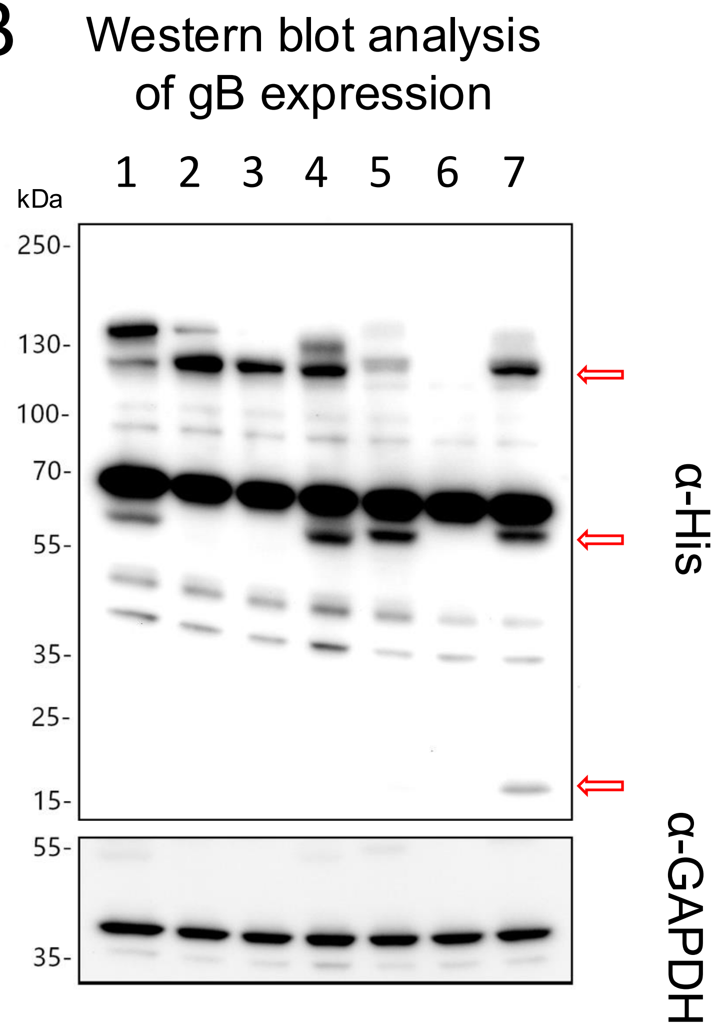

C

1: gB_KSHVecd-RRVtm-RRVicd-His in pCAGGS (AX681)

2: RRVecd-KSHVtm-KSHVicd-His in pCAGGS (AX682)

3: RRVecto-RRVtm-KSHVicd-His in pCAGGS (AX683)

4: KSHV gB-His in pCAGGS (MB157)

5: RRV IGG2SP N-term Strep gB-His c.o. in pCAGGS (MB148)

6: Empty pCAGGS

7: RRV gB in pcDNA6V5His (AX223)

ECD: extracellular domain (KSHV aa 1-732, RRV aa 1-717) ; TM: transmembrane domain (KSHV aa 733-752, RRV aa 718-737); ICD: intracellular domain (KSHV aa 753-845, RRV aa 738-829); c.o.: codon optimized; His: 8 histidine tag; IGG2SP: IgG2 signal peptide; N-term: N-terminal; Strep: tandem Strep tag II (WSHPQFEK-GGGSGGGSGGSA-WSHPQFEK)
