# Supplementary material for: Kaposi’s sarcoma-associated herpesvirus (KSHV) gB dictates a low-pH endocytotic entry pathway as revealed by a dual-fluorescent virus system and a rhesus monkey rhadinovirus expressing KSHV gB
Source: PLoS Pathog. 2025 Jan 16;21(1):e1012846. doi: 10.1371/journal.ppat.1012846 (PMC11801733; doi:10.1371/journal.ppat.1012846)
Supplement: S1 Fig — (A) Fusion activity of different KSHV and RRV gB variants co-expressed with RRV gB in 293T effector cells with EphA2-overexpressing Raji target cells. **** p<0.0001; ordinary one-way ANOVA, Dunnett test for multiple comparison. All values were log-transformed prior to analysis. (B) Expression of different KSHV and RRV gB variants in transfected 293T cells. The numbering corresponds to the legend in (A). The Western blot was detected with an anti-His-Tag antibody at 1:1000 dilution (mouse monoclonal, clone 4E3D10H2/E3, Invitrogen) and anti-mouse HRP-coupled secondary antibody. Arrows indicate gB-specific reactivities. Constructs 1–5 use pCAGGS as a vector backbone, construct 6 pcDNA6V5His. Plasmid MB148 (IgG2 signal peptide followed by N-terminal tandem Strep tag II (WSHPQFEK-GGGSGGGSGGSA-WSHPQFEK) fused via an Ser-Arg encoding XbaI linker to RRV gB amino acids 26–829 codon optimized) was synthesized/constructed by Genscript using accession AAC58686.1 as template and pCAGGS as backbone. The KSHV gB coding sequence in AX223 was directly cloned from RRV-YFP (accession MN488839.2) into the pcDNA6V5His backbone. Plasmids MB113 (IgG2 signal peptide followed by N-terminal tandem Strep tag II fused via an Ser-Arg encoding XbaI linker to KSHV gB codon optimized), MB137 (IgG2 signal peptide followed by N-terminal tandem Strep tag II WSHPQFEK-GGGSGGGSGGSA-WSHPQFEK fused via an Ser-Arg encoding XbaI linker to KSHV gB codon optimized amino acids 27–845, C-terminal His-tag), MB157 (KSHV gB, C-terminal His-tag) and MB158 (KSHV gB L791A L792A Y830A, C-terminal His-tag) were synthesized/constructed by Genscript (accession MK143395.1 as template) using pCAGGS as backbone. c.o. = codon optimized. (C) 293T cells were transfected with the indicated constructs and surface expression was determined using 7C2 anti-gB antibody (hybridoma supernatant) or a control hybridoma supernatant. The experiment was repeated three times. The gBΔ58 construct was obtained by PCR mutagenesis of plasmid MB [file ppat.1012846.s002.pdf]

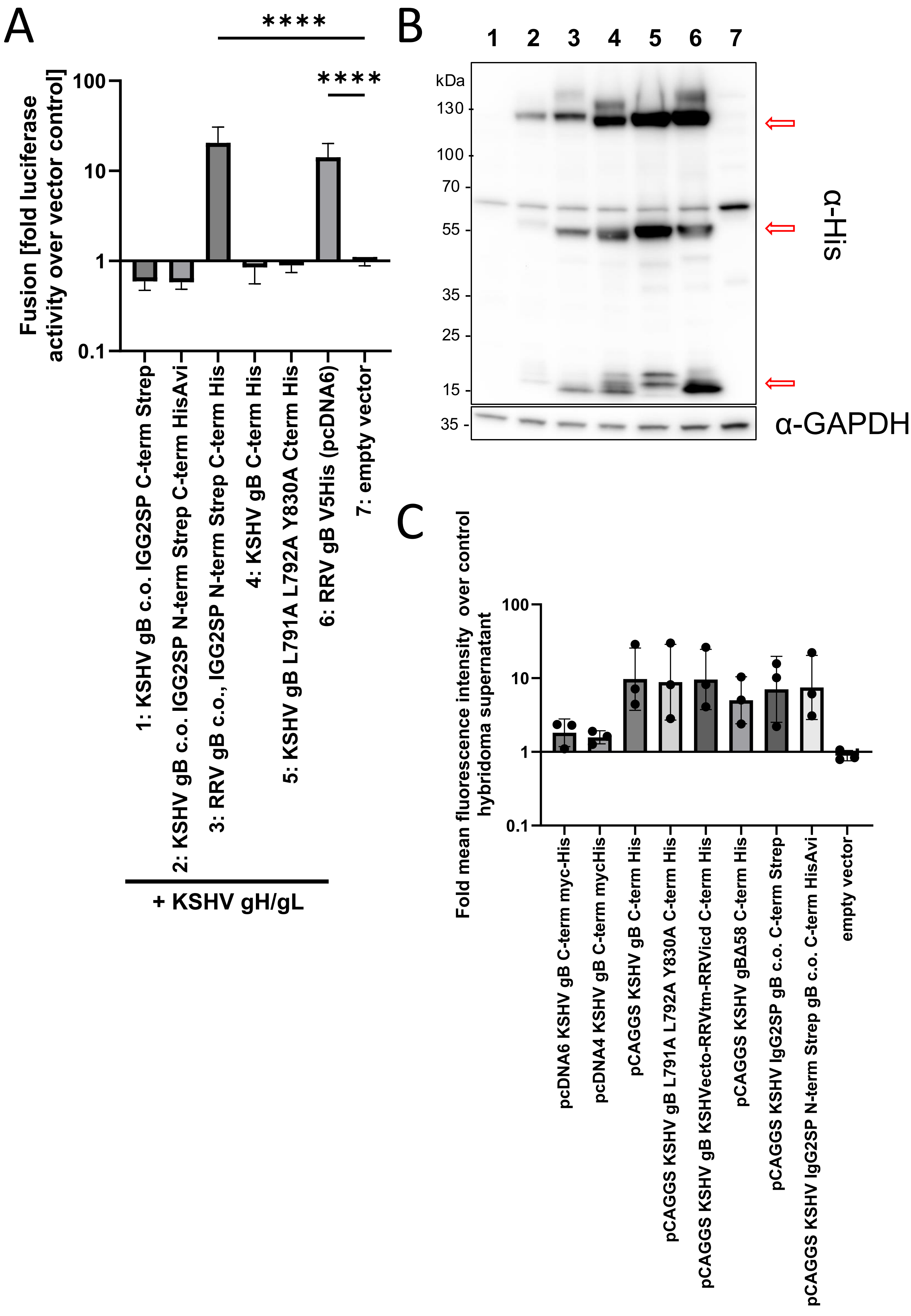
A
B
C
****
****
Fusion [fold luciferase activity over vector control]
100
10
1
0.1
1: KSHV gB c.o. IGG2SP C-term Strep
2: KSHV gB c.o. IGG2SP N-term Strep C-term HisAvi
3: RRV gB c.o., IGG2SP N-term Strep C-term His
4: KSHV gB C-term His
5: KSHV gB L791A L792A Y830A Cterm His
6: RRV gB V5His (pcDNA6)
7: empty vector
+ KSHV gH/gL
1 2 3 4 5 6 7
kDa
130
100
70
55
35
25
15
35
α-His
α-GAPDH
Fold mean fluorescence intensity over control hybridoma supernatant
pcDNA6 KSHV gB C-term myc-His
pcDNA4 KSHV gB C-term mycHis
pCAGGS KSHV gB C-term His
pCAGGS KSHV gB L791A L792A Y830A C-term His
pCAGGS KSHV gB KSHVecto-RRVtm-RRVicd C-term His
pCAGGS KSHV gBΔ58 C-term His
pCAGGS KSHV IgG2SP gB c.o. C-term Strep
pCAGGS KSHV IgG2SP N-term Strep gB c.o. C-term HisAvi
empty vector
